# Step Test for Rapid Screening of Material and Process Parameters for Resin Development in DLP 3D Printing

**DOI:** 10.1002/anie.202504154

**Published:** 2025-07-06

**Authors:** Michelle Vigogne, Cosima Aeschbach, Ricardo Bernhardt, Anika Kaufmann, Julian Thiele

**Affiliations:** ^1^ Leibniz‐Institut für Polymerforschung Dresden e.V. Hohe Straße 6 01069 Dresden Germany; ^2^ Institute of Chemistry Otto von Guericke University Magdeburg Universitätsplatz 2 39106 Magdeburg Germany

**Keywords:** Biobased photoinitiator, DLP 3D printing, Parameter screening, Renewable materials, Step test

## Abstract

The success of 3D printing based on vat polymerization by spatially controlled UV light exposure strongly depends on appropriate resins. However, in search of new resins, optimizing a resin's composition as well as its printing parameters can be time‐consuming and labor‐intensive. To address these challenges, we present a rapid parameter screening that enables efficient evaluation of 24 printing parameter settings within one print focusing on exposure energy, exposure time, and layer thickness as the most crucial process parameters in 3D printing based on digital light processing (DLP). Our step test minimizes the required number of experimental iterations and 3D prints by relying on optical characterization without the need for special analytical devices, making it rapid and accessible for a broad range of users across different disciplines. For a proof of concept, the step test is applied to define optimal printing parameters of three commercial resins and evaluate the polymerization efficiency of two predominantly biobased, noncommercial resins containing either phenylbis(2,4,6‐trimethylbenzoyl)phosphine oxide or curcumin as photoinitiators. With that, we introduce a straightforward test that is easy to implement and adaptable to other UV polymerization‐based printing methods, speeding up the time from resin screening to optimal printing parameters and products.

## Graphical abstract

We present an efficient and rapid step test for determining optimum printing parameters in digital light processing (DLP). It enables the testing of 24 printing parameter settings within one print, is easy to implement, and adaptable to other UV polymerization‐based printing methods, thus speeding up the time from resin screening to optimal printing parameters and products. Yet, it only involves simple validation by eye of 3D‐printed test structures.

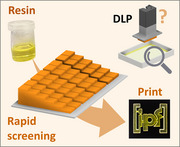

## Introduction

Digital light processing (DLP) has become an increasingly important 3D printing process in recent years with various applications in fast prototyping,^[^ ^1^, ^2^, ^3^, ^4^ ^]^ dentistry,^[^ ^5^, ^6^, ^7^, ^8^ ^]^ microfluidics,^[^ ^9^, ^10^, ^11^, ^12^, ^13^, ^14^, ^15^ ^]^ and sensor technology,^[^ ^16^, ^17^, ^18^ ^]^ particularly due to user‐friendliness, freedom of design, and precision of the printing process.^[^ ^19^, ^20^ ^]^ With the emerging use of DLP 3D printing, the focus of resin development has moved toward green and sustainable materials and components.^[^ ^21^, ^22^ ^]^ Among these, isobornyl acrylate (IBOA), a biomass‐derived terpene component that does not compete with food production, is one of the few commercially available biobased monomers already being explored for industrial applications as well as for DLP 3D printing.^[^ ^23^, ^24^, ^25^ ^]^ Furthermore, riboflavin, curcumin, coumarin, or flavone have previously been studied^[^ ^26^, ^27^, ^28^, ^29^ ^]^ to replace conventional photoinitiators like phenylbis(2,4,6‐trimethylbenzoyl)phosphine oxide (BAPO),^[^ ^30^, ^31^, ^32^ ^]^ diphenyl(2,4,6‐trimethylbenzoyl)phosphine oxide (TPO),^[^ ^31^, ^32^ ^]^ or Ivocerin.^[^ ^31^, ^33^ ^]^ Wendland et al. focused on curcumin and riboflavin as type II photoinitiators with various biobased co‐initiators like lysine, arginine, or histidine. Notably, curcumin can also react without an additional co‐initiator, simplifying the chemical composition of the resin.^[^ ^29^ ^]^

When investigating any new resin, determining printing parameters is still time‐consuming and commonly associated with trial‐and‐error experiments as there are only a few automated processes.^[^ ^34^, ^35^ ^]^ This applies especially for DLP 3D printing, which depends on many factors, ranging from the composition of resin formulation in terms of monomer, crosslinker, photoinitiator, and additives that affect the resin's reactivity, to the influence of the printing parameters such as layer height, exposure energy, layer orientation, and postprocessing of 3D‐printed objects.^[^ ^32^, ^36^ ^]^ Commonly, the cured polymer layer height is evaluated as a function of the exposure energy, yielding the so‐called Jacobs workings curves.^[^ ^37^ ^]^ These can be used to derive parameters such as the penetration depth of the UV light and the critical exposure energy for the polymerization. However, such investigations are time‐consuming, often carried out manually, and are only an approximation of the printing parameters as the test is not performed in the printing process mode but simply based on the exposure of resin spots on a glass slide.^[^ ^12^, ^38^, ^39^, ^40^, ^41^, ^42^ ^]^ To further shed light on the photopolymerization process and resin solidification, respectively, ATR‐FTIR spectroscopy has been utilized to reveal the conversion of double bonds.^[^ ^35^, ^43^ ^]^ In addition, UV curing behavior has been studied using photorheology.^[^ ^35^, ^44^ ^]^ However, using these methods does not reveal which printing parameters are required to achieve sufficient adhesion of a polymerized resin to the build platform, or what exposure energy is needed for different resin layer thicknesses, e.g., from thin layers for high‐resolution prints to thick layers for rapid prototyping. It would be highly beneficial if exactly these parameters could be better determined through an easily accessible, automated printing process. In a first attempt, Choi et al. developed an iterative and multistage process for determining printing parameters, starting with a single‐layer test to create Jacobs working curves, followed by a resin‐recoating test to evaluate resin viscosity, an adhesion test to determine printing parameters for the very first layer at the build platform, and another test to avoid over‐curing.^[^ ^34^ ^]^ Kowsari et al. investigated the influence of print quality and precision compared to the underlying CAD model at different exposure energies and for different resin formulations by means of scanning electron microscope (SEM) measurements, a high‐resolution but time‐consuming method,^[^ ^45^ ^]^ which requires expensive equipment. In addition to the lateral resolution, where over‐curing should be avoided in particular, and the analysis of resin reactivity, the layer‐to‐layer adhesion is another crucial factor for DLP 3D printing that is influenced by the total exposure energy. Here, a low exposure energy can lead to inadequate bonding between layers and thus defects such as delamination, micro‐sized interlayer defects, and reduced mechanical stability.^[^ ^8^, ^46^ ^]^

## Results and Discussion

**Figure 1:**
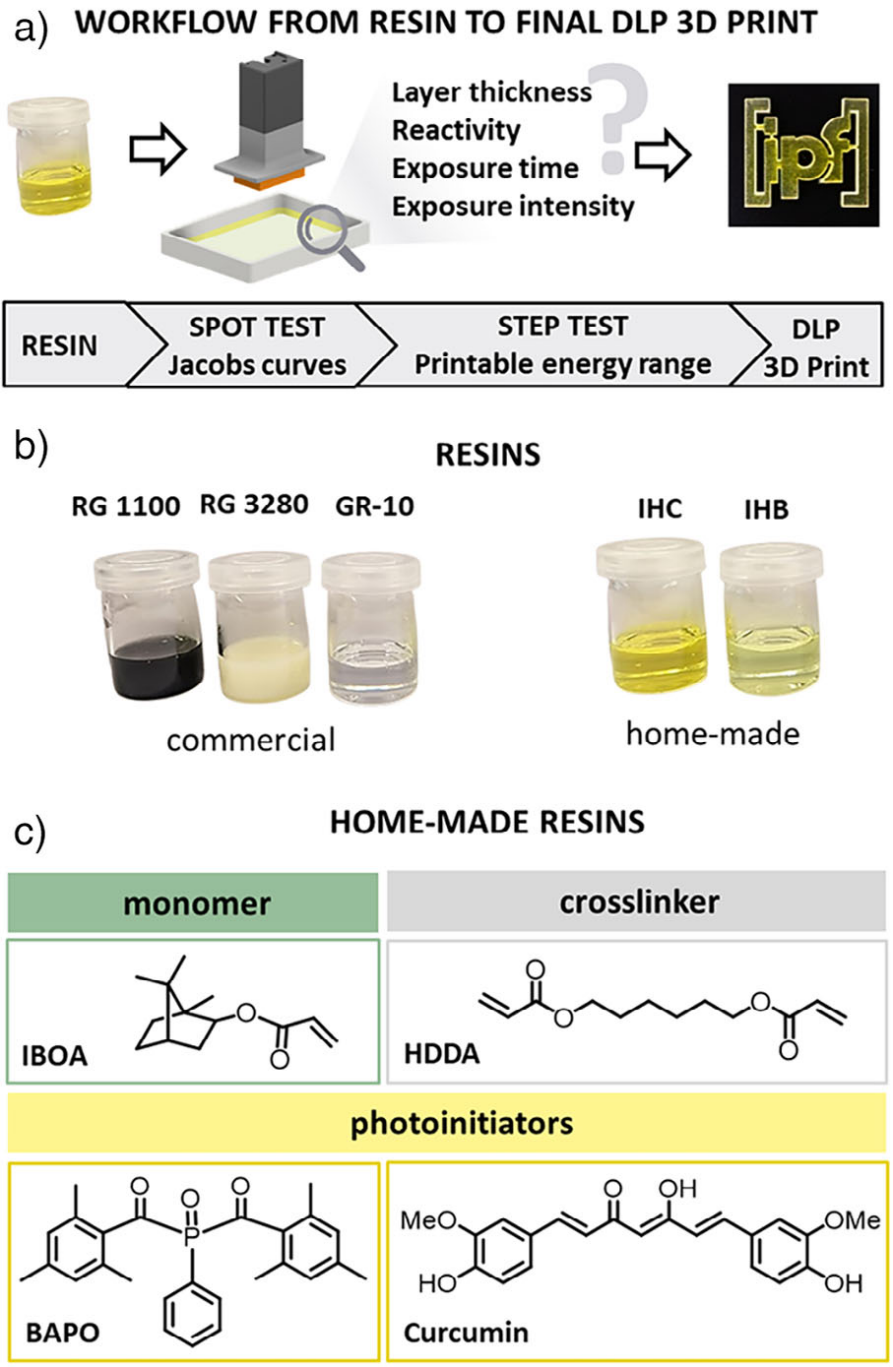
a) Workflow from liquid resin to final DLP 3D print. b) Images of commercial (RG 1100 Black, RG 3280, GR‐10 guide) and home‐made (IHC, IHB) resins. c) Chemical structures of resin components for IHB and IHC containing **I**BOA as monomer, **H**DDA as crosslinker, and **B**APO or **c**urcumin as photoinitiator.

We present a rapid step test for screening 24 parameter combinations within one 3D print that can be easily applied for new material development in DLP 3D printing. We integrated our newly developed step test into the general workflow for resin development, starting with a spot test to determine Jacobs working curves and specify the required exposure energy range, followed by the step test to define optimal printing parameters and printing a 3D object with these parameters (Figure 1a). We applied our test routine to three commercial resins, namely, Ultracur3D RG 1100 Black and Ultracur3D RG 3280 from BASF as well as Printodent GR‐10 guide from Pro3Dure, and two home‐made resins (Figure 1b) to address both user groups: those using 3D printing as a manufacturing and prototyping technique and those developing new resins to address specific needs, e.g., structure resolution on the micrometer‐scale. The home‐made resins presented in here contain IBOA as biobased monomer and conventional 1,6‐bis(acryloyloxy)hexane (HDDA) as crosslinker. Two different photoinitiators, namely, BAPO (resin formulation IHB) and curcumin as biobased alternative (resin formulation IHC), were tested regarding their polymerization efficiency (Figure 1c, cf. Table S1).

### Spot Test for Jacobs Working Curves

The analysis of exposure energy in correlation to the resulting cure depth (*C*_d_) is a well‐known method for obtaining process‐relevant parameters for DLP 3D printing. For this purpose, Jacobs workings curves (Equation 1) are analyzed to determine information such as the penetration depth of UV light into resin (*D*_p_) and the critical exposure energy (*E*_c_) for photopolymerization by correlating *C*_d_ and ln *E*.^[^ ^37^ ^]^

(1) $$C_{d}=D_{p}\;\ln\left(\frac{E}{E_{c}}\right)$$

Here, the spot test consists of five projection layers, where three circular spots are exposed by UV light and assigned to one parameter set per 3D‐printed layer. In this way, five exposure energies (*E*_1_–*E*_5_) are examined in triplicate per 3D print by varying the exposure time (*t*_1_–*t*_5_) and the set exposure intensity (*I*) (Figure S1a,b). This preliminary spot test is used to estimate a resin's potential exposure energy for DLP 3D printing and provide information about its reactivity as well as achievable layer thicknesses, which can also be influenced by factors like photoabsorption. As photoabsorbers increase *E*_c_ and decrease *D*_p_, they allow control of *C*_d_ and *z* axis resolution when printing microchannel structures or overhangs.^[^ ^44^, ^47^ ^]^As for spot tests only a small amount of resin is required, it is a material‐efficient method to initially obtain a sufficient approximation of printing parameters. For the three commercial resins, the Jacobs working curves provided information about the individual reactivity of each resin, which followed the trend RG 3280 > RG 1100 Black > GR‐10 guide (cf. Figure S1c). All three resins exhibited a low *E*_c_ value of less than 10 mJ cm^−2^ (*E*_c, RG1100 Black_ = 4.4 mJ cm^−2^, *E*_c, RG3280_ = 0.4 mJ cm^−2^, *E*_c, GR‐10 guide_ = 6.2 mJ cm^−2^) and can therefore also be used with DLP 3D printers with a low exposure intensity. The determined penetration depths *D*_p_ showed that UV light penetrated deeper into the transparent GR‐10 guide (*D*_p_ = 210 µm) than into RG 1100 Black (*D*_p_ = 134 µm), whereas the ceramic particle dispersion RG 3280 revealed the lowest UV light propagation with a *D*_p_ of 81 µm (cf. Table S3, Figures S2–S4).

For the home‐made resins IHB and IHC, no linear correlation between *C*_d_ and ln *E* was obtained, so *E*_c_ and *D*_p_ could not be determined from the Jacobs curves (cf. Figures S5–S7). However, due to the very low *C*_d_ of IHC at a considerably higher exposure energy of 1800–7200 mJ cm^−2^ than IHB, which is exposed with 50–900 mJ cm^−2^, it can already be assumed that the polymerization efficiency of BAPO as a photoinitiator will be higher than that of curcumin. These results indicate that the spot test is a valuable method for fast curing materials with a fast transition between non‐crosslinked state and fully cured state as shown here for RG 3280, RG 1100 Black, and GR‐10 guide, but error‐prone for slow curing systems with reduced crosslinking density such as IHB and IHC.

Disadvantages of the spot test as a single method for resin characterization are:

1. It is only carried out on a glass slide and thus outside a real printing process.^[^ ^34^ ^]^
2. It is time consuming due to manual height measurement.
3. It does not provide information about the printing accuracy at a certain exposure energy.
4. It is prone to errors for resins that are soft, elastic, or slow curing systems as errors in *C*_d_ affect the calculation of *E*_c_ and *D*_p_.^[^ ^38^, ^44^ ^]^

### Step Test for Rapid Parameter Screening

**Figure 2:**
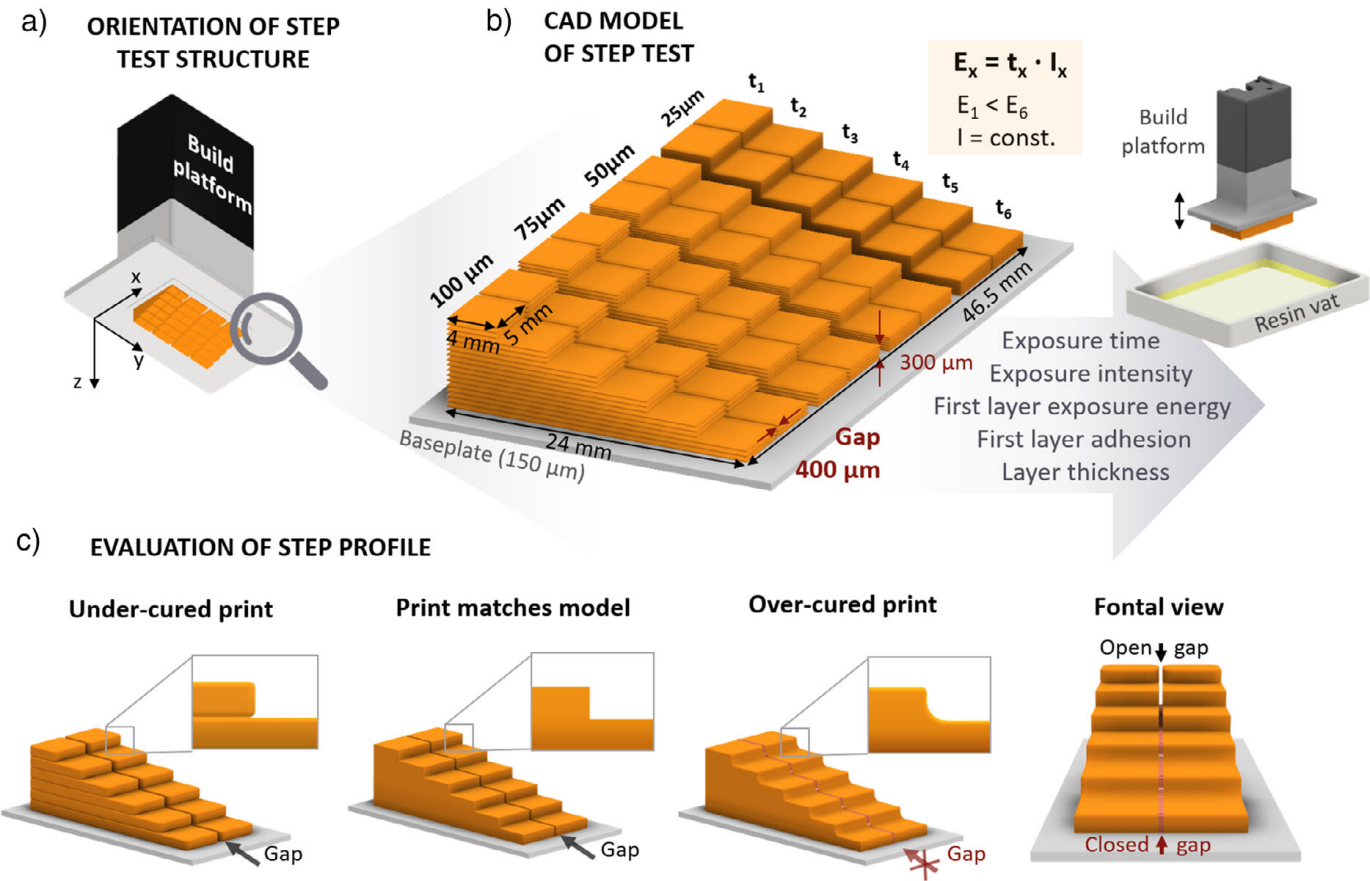
CAD model of the newly developed step test (4‐fold scaling in *z* direction for improved visualization). a) Step test structure at the build platform to visualize the printing orientation. b) Dimensions of the four staircases for testing four layer heights (25, 50, 75, 100 µm) with six steps (each 300 µm) for testing six exposure times; each staircase is separated by a gap of 400 µm for further evaluation. c) Optical validation of printing parameters using the step profile: under‐cured (incomplete, delaminating, or missing layer), printed as provided by the CAD model, and over‐cured (gap between stairs closed).

With our novel step test, we can overcome the limitations of the spot test as the printing is performed directly on the build platform and thus simulates the real conditions during printing and provides information about parameters such as the exposure energy for a selected layer thickness. By combining six exposure times with a constant exposure intensity during the print, six exposure energies (E) can be screened (E_1_ < E_6_) for four layer thicknesses resulting in a total of 24 combinations, which can be tested with a single 3D print. For this approach, a six step‐shaped object is developed where each physical step represents a combination of different layer thicknesses and exposure times with increasing exposure time (*t*) from top to bottom (*t*_1_ < *t*_6_) for each step (Figure 2a, cf. Figure S8). This means that steps closer to the build platform are printed with longer exposure times to ensure a good adhesion to the printing platform, thus preventing early defects in the 3D‐printed structure due to delamination of layers or missing layers due to under‐curing, meaning insufficient crosslinking of the photopolymer network. For rapid visual validation of the printing parameters, we include a 400 µm gap in the step test 3D model in each of the four staircases (Figure 2b). To ensure sufficient adhesion between the printed step test object and the build platform, a baseplate being 150 µm in thickness, meaning three 50 µm layers, is printed onto the build platform with the initial *E*_6_ as exposure energy (Figure 2b). This baseplate made of the resin to be investigated can be used to determine which exposure energy is required for the first layer and whether the material adheres to the build platform. If this is not the case, the baseplate can be printed with an adhesion layer, using a resin which is known to have an adequate adherence to the surface of the printing platform.

Here, the size of the step test model was set for an ASIGA MAX X 3D printer with a lateral building area of 29 x 51 mm (*x*, *y*), but the model can be easily adapted and customized, e.g., it can be adjusted to any other printer with its specific building area or the number of steps can be varied. A prerequisite for adapting the step test to other DLP 3D printers is a software environment that allows the print to be subdivided into different printing ranges to which different printing parameters can be assigned. This complete parameter control enables a systematic definition of the process parameters. For higher resolution testing or highly absorbing resins, the gap size or the layer thickness can be reduced, with the selected layer thickness always being a multiple of the lowest layer thickness. It is worth mentioning that the printing parameters are determined individually for each layer thickness as the exposure intensity decreases during the propagation through the resin due to the Beer–Lambert relationship.^[^ ^35^, ^38^ ^]^

Initial light intensity and exposure times are provided by the Jacobs workings curves determined by the spot tests. By varying the exposure time and intensity, the tested exposure energy range is defined according to the correlation *E*_x_ = *t*_x_ × *I*_x_. The initial exposure intensity is selected based on where stable *C*_d_ values of approx. 100 µm have been achieved. The selectable light intensity depends on the properties of the used DLP 3D printer. In addition, the spot test can be used to estimate whether the resin is a fast or slow curing system. Depending on the reactivity of the resin, a time interval between short exposure times and long exposure times can be selected as higher exposure energies lead to better layer‐to‐layer adhesion, whereas low exposure energies tend to lead to incomplete layer formation or delamination and thus print defects in form of missing steps or even structure collapse. Consequently, the step test starts with the longest exposure time (*t*_6_) and ends with the shortest exposure time (*t*_1_) in fixed increments.

The validation of printing parameters from the printed step test is carried out by simple optical evaluation of the step profile without the use of elaborate or expensive analytical tools and methods. For assessing the desired printing parameter set of layer thickness and exposure energy, the individual steps are rated with 1 for steps with good printing quality and 0 for insufficiently printed steps. For a rating of 1 or “good”, the following quality criteria are required: 1) a stable step with sharp edges, 2) an open 400 µm gap so that the print is in good agreement with the CAD, and 3) a mechanically stable surface finish that cannot be easily scratched or damaged. A rating of 0 is used for those steps that exhibit under‐curing, delamination of layers, unstable and sticky layers, or missing layers (“under”), or the opposite that means strong over‐exposure, resulting in over‐curing (“over”), which can be easily identified visually by the 400 µm gap being closed. A continuous open gap between two staircases enables the user to differentiate the print quality of each individual step. To do this, the user can visually evaluate the step directly from the front of the step test structure, focusing on the gap. This allows the user to assess whether lower layers with longer exposure times are over‐cured and upper layers with shorter exposure times show the desired print quality (Figure 2c).

**Figure 3:**
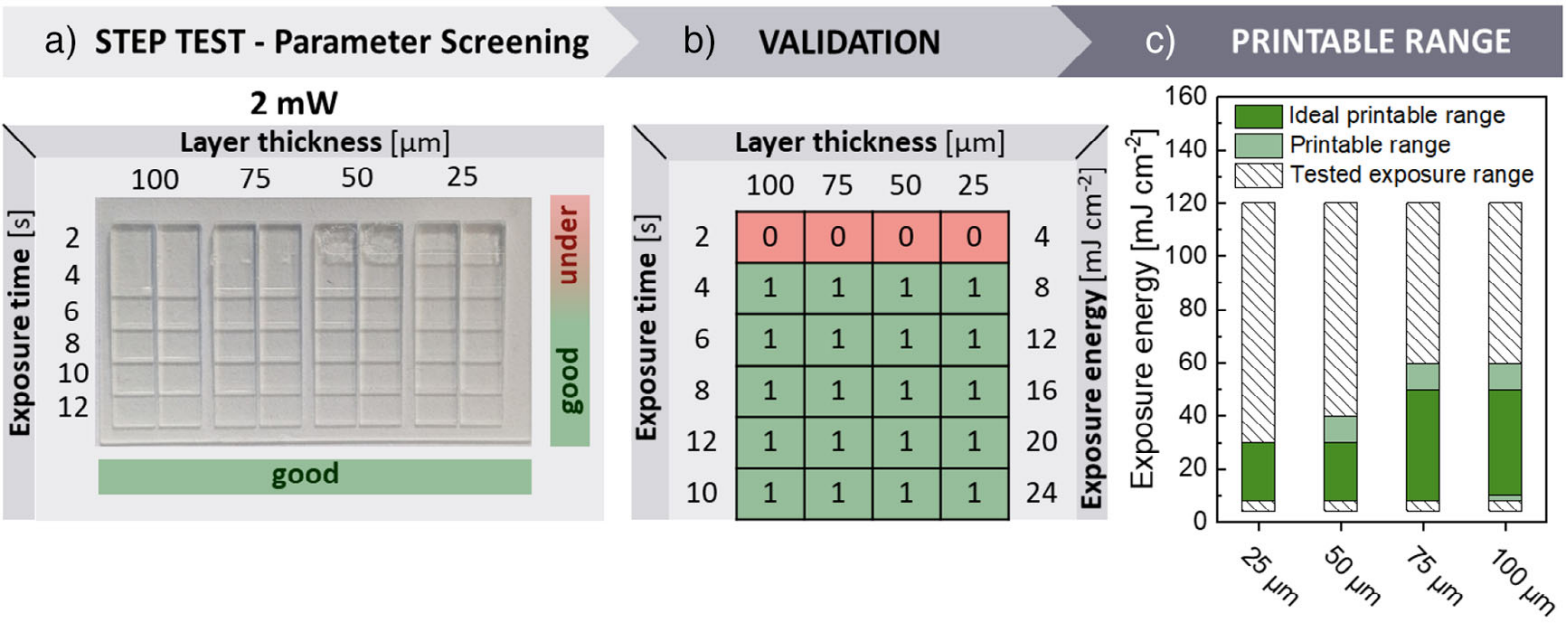
Determination of the printable range of the commercial resin GR‐10 guide via the step test; a) image of 3D‐printed steps at 385 nm with 2 mW exposure intensity and 2–12 s exposure time. Rating of the steps with “good” and “under” by visual validation by eye, b) rating of all 3D‐printed steps with “1” or “0” for matrix analysis. c) Overview on the tested exposure range and validated printable range for layer thicknesses between 25–100 µm (ideal printable range: 100% success rate, printable range: >49% success of the printing parameter obtained from the 3‐fold replication).

Exemplarily, we performed the step test for the three commercial resins and the received printing parameters were compared with the printing parameters given by the manufacturer. To verify whether the quick visual validation by eye is sufficient or a further examination is necessary, the step profiles for GR‐10 guide were examined additionally via reflected light microscopy. For that, 96 steps of four prints with each 24 steps were assessed for different exposure intensities to check for sharp edges of steps, open or closed gaps between stairs, and any over‐curing in lateral direction. Here, the visual validation by eye agreed with reflected light microscopy analysis in 93 of the 96 analyzed steps with a slight over‐curing could be detected in three cases, which was not apparent by eye (Figure 3a,b, cf. Figures S9b and S11). Due to the reliable visual analysis verified by reflected light microscopy, the much faster visual inspection can be used in all following experiments. To define a suitable energy range for the step test, a time interval with six different exposure times is selected, e.g., *t*_1_–*t*_6_ = 1–6 s, *t*_1_–*t*_6_ = 2–12 s, *t*_1_–*t*_6_ = 5–30 s, or *t*_1_–*t*_6_ = 10–60 s and tested with a continuous exposure intensity per step test (cf. Table S2). The upper limit is determined by the occurrence of over‐curing, whereas the lower limit is reached when the exposure intensity is so low that under‐curing results in inadequately printed steps. Typically, three to four step tests with different exposure intensities are required to accurately determine these limits.

For analyzing the printing parameters of the resin GR‐10 guide, four step tests with exposure intensities of 2, 5, 10, and 20 mW (cf. Figure S9), presenting an exposure energy range from 4 to 120 mJ cm^−2^, were printed. To confirm its reliability, a threefold replication of the step test with each exposure energy was performed. Afterward, a success rate (%) was calculated based on the threefold replication. This success rate indicates whether the respective printing parameter could be printed successfully in one of three prints (33%), in two of three prints (67%), or in three of three prints (100%). This information was then inserted in the exposure energy diagrams, which are divided into three parts: tested energy range, printable range (success rate >49%), and ideal printing range (success rate 100%). By analyzing the step quality, an ideal printable range was determined for 25 and 50 µm layers with an exposure energy of 8–30 mJ cm^−2^, for 75 µm layers with an exposure energy of 8–50 mJ cm^−2^, and for 100 µm layers with an exposure energy of 10–50 mJ cm^−2^ (Figure 3c). In addition to the total exposure energy, the combination of exposure time and exposure intensity also influenced the printing result. For example, if a 50 µm layer was exposed with an energy of 40 mJ cm^−2^, it had a good printing quality at 5 mW and 8 s, as well as when the exposure time is halved to 4 s and the intensity increased to 10 mW. However, a further increase in the exposure intensity to 20 mW with an even shorter exposure time of 2 s was already sufficient to induce over‐curing (cf. Figure S9). Therefore, it is crucial to test the same exposure times across different exposure intensities to determine both the energy range and the minimum and maximum exposure times for a given intensity. These results could be visualized graphically to provide an overview of suitable exposure times in relation to the exposure energy and to show which layer thicknesses could be successfully printed (Figure S10). In addition to a swift inspection of the 3D‐printed step structure by eye as well as reflected light microscopy, the printing accuracy was confirmed using SEM. For that, squares with a size of 540–1080 µm and a layer height of 25 µm were 3D‐printed with GR‐10 guide at 8 mJ cm^−2^, depicting the lower end of the printing range; at 20 mJ cm^−2^, being the middle of the printing range, and at 40 mJ cm^−2^, which represented the upper end of the printing range (cf. Figure 3c). The printing accuracy for 8 mJ cm^−2^ was between 0.94 and 0.99, representing a slight under‐curing; for 20 mJ cm^−2^, between 1.00 and 1.04, and for 40 mJ cm^−2^, between 1.03 and 1.07, indicating slight over‐curing (cf. Figure S12). Nevertheless, these deviations are minimal, and the printed objects are in good agreement with the CAD model. This demonstrates that the printing parameters obtained by the step test and assessed by eye resulted in a printing accuracy between 0.94 and 1.07 determined by SEM, highlighting the ease of use of the step test and also expanding the potential user group due to its time efficiency and simplicity.

Finally, the now‐defined printable range was compared to the printing parameters provided by the manufacturer. The exposure energy for the biocompatible and transparent dental resin GR‐10 guide is 11.8 mJ cm^−2^ for 25 µm layers as well as 16.5 mJ cm^−2^ for 100 µm layer thickness (ASIGA MAX X 385 nm), as obtained from the ini.files of the manufacture Pro3Dure.This is in very good agreement with the results of our step tests, which determined a printable range with exposure energies of 8–30 mJ cm^−2^ for 25 µm layers and 10–50 mJ cm^−2^ for 100 µm layers.

For a second commercial resin, RG 1100 Black, we performed three step tests to cover an exposure energy range from 4 to 120 mJ cm^−2^, where under‐curing occurred at 2 mW, good print quality was achieved at 5 mW, and over‐curing was reached after a further intensity increase to 10 mW (cf. Figure S13). Based on this approach, the ideal printable range was 8–24 mJ cm^−2^ for 25 µm, 8–30 mJ cm^−2^ for 50 µm layers, 8–40 mJ cm^−2^ for 75 µm layers, and 8–50 µm for 100 µm layers (cf. Figure S15). The printable range for 100 µm layers was in good agreement with the given exposure energy from the manufacturer's user guideline made public for different printers, namely, 20.25 mJ cm^−2^ (Miicraft Ultra 125 Y), 12.5 mJ cm^−2^ (Stratasys Origin one), and 10 mJ cm^−2^ (Rapidshape i30+), respectively, each determined with a DLP 3D printer operating at 385 nm. Manufacturers' data sheets primarily specify exposure energy values for 100 µm layers for different printers and wavelengths as these represent a compromise between printing time and resolution, which is particularly important for printing larger objects in industrial applications. For process validation, fabricated parts are often controlled by mechanical characterization. Therefore, RG 1100 Black was exemplary investigated. The resin was printed with an exposure energy of 17.5 mJ cm^−2^ for 100 µm layers, which is within the printable range determined by the step test and was also used by the manufacturer to investigate the mechanical values from tensile and bending tests. The measured values are in good agreement with the values given by the resin manufacturer in brackets: the E modulus is 3149 ± 480 MPa (2,950 MPa), the tensile strength is 60 ± 1 MPa (70 MPa), the elongation at break is 6 ± 1% (5%), the flexural modulus is 2707 ± 182 MPa (2,790 MPa), and the flexural strength is 99 ± 11 MPa (125 MPa) (cf. Table S4). For a third commercial resin, RG 3280, which is a high‐temperature material with white color, four step tests were performed, covering an exposure energy range of 1–60 mJ cm^−2^. As RG3280 is a fast‐curing resin, the first step tests with 2 and 5 mW were already over‐cured and the intensity had to be further reduced to 1 mW and exposure times shortened to define the lower limit (cf. Figure S14). The validation of the step tests yielded a printable range of 1–2 mJ cm^−2^ for 25 µm layers, 1–3 mJ cm^−2^ for 50 µm layers, 2–5 mJ cm^−2^ for 75 µm, and 2–6 mJ cm^−2^ for 100 µm layers. Overall, the resin had a very high reactivity, causing strong over‐curing even at 2 mW. The obtained printable range for 100 µm layers (cf. Figure S15) was comparable with the exposure energy found in a user guideline of BASF made public for different printers at 385 nm, namely of 4 mJ cm^−2^ (Miicraft Ultra 125 Y) and 7 mJ cm^−2^ (Stratasys Origin one), respectively. These results show that our step test can be used to rapidly and reliably investigate a resin and determine its exposure energy for a specific layer thickness, which is in good agreement with printing parameters specified by the resin manufacturer, such as layer thickness, exposure time, exposure intensity, and wavelength. Based on the results of standardized process parameter screening coupled with mechanical characterization of components by tensile and/or bending testing, it is reasonable to discuss the application of our step test beyond laboratory scale. Indeed, industrial workflows likewise lack efficient identification of process parameters and printing conditions, which has become a bottleneck in additive manufacturing. Being rapid without requiring costly analytical equipment and applicable to a variety of commercial resin types, the step test fulfils key requirements of a time‐ and cost‐effective industrial workflow. Here, the threefold replication provides sufficient reliability of printing parameters even for material development or processing.^[^ ^48^ ^]^ At the same time, a DIN standard for DLP 3D printing parameter screening has not been implemented yet. Exemplarily, in 2018, a standards committee “Additive Manufacturing Processes Advisory Board” was set up,^[^ ^49^ ^]^ and in 2019, first requirements for quality‐assured processes were published, which define uniform requirements for additive manufacturing (DIN SPEC 17071:2019 and DIN EN ISO/ASTM 52920:2023).^[^ ^50^, ^51^ ^]^ However, the development of standardized methods for determining process parameters for individual printing techniques is still ongoing.

Further trends can be derived from the step tests regarding the minimum exposure times for a given resin as well as realizable layer thicknesses between 25 and 100 µm at a given exposure energy. For example, as the absorption of a resin increases, the penetration depth of UV light into the resin decreases, which reduces the realizable layer thicknesses as well as the required exposure energy. In addition, the choice of layer thickness has a direct effect on the print quality. A higher layer thickness generally requires a higher exposure energy to ensure complete curing of the layer, whereas narrow layers tend to over‐cure. Looking at the printable range depending on the layer thicknesses offers the advantage that parameters for high‐resolution prints with low layer heights as well as fast prototyping with high layer heights can be determined in one print.

**Figure 4:**
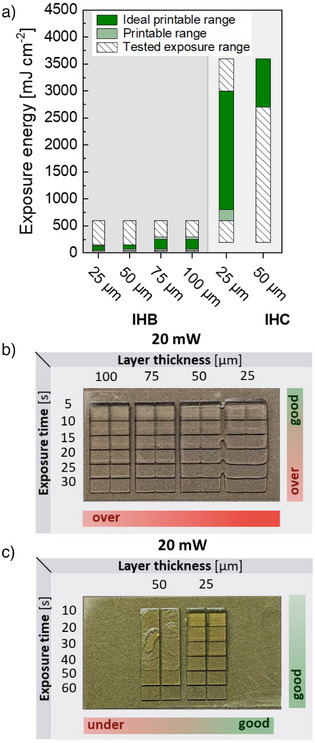
a) Overview on tested exposure range and printable range of home‐made resins IHB and IHC as determined by the step test at 405 nm. For IHC, a layer thickness of 75 µm was only printable with 3600 mJ cm^−2^, whereas 100 µm could not be obtained (ideal printable range: 100% success rate, printable range: >49% success of the printing parameter obtained from the 3‐fold replication). Exemplary images of 3D‐printed step tests and rating of step quality to determine the printable range of home‐made resins b) IHB and c) IHC.

To expand the view on potential resin formulations that may benefit from the step test, we also demonstrate the rapid determination of printing parameters for home‐made resins. In the field of material development for DLP 3D printing, there is a clear trend to transition from petrochemical to biobased materials.^[^ ^21^, ^22^ ^]^ For this reason, resins consisting of IBOA, HDDA, and two different photoinitiators were tested. Due to the low solubility of curcumin in IBOA and HDDA (approx. 0.2–0.5 wt%), 0.2 wt% of curcumin (resin IHC) or BAPO (resin IHB), respectively, was used as photoinitiator to ensure complete solubility and comparability. After dissolving the photoinitiator, IHC exhibited a very strong yellow color, whereas IHB showed only a light‐yellow color. By changing the photoinitiator from BAPO to curcumin, the reactivity of the resin decreased, resulting in a shift of the ideal printable range for 25 µm layer thickness from 50–125 mJ cm^−2^ for IHB to 800–3000 mJ cm^−2^ for IHC, and for 50 µm layer thickness from 75–150 mJ cm^−2^ for IHB to 2700–3600 mJ cm^−2^ for IHC (Figure 4, cf. Figure S16). Due to the low reactivity of curcumin as a photoinitiator, only layer thicknesses of 25–50 µm could be reliably printed in the exposure energy range given by the used 3D printer (cf. Figure S17). In the initial spot test, only 10 µm cure depth was achieved for IHC between 1800 and 3600 mJ cm^−2^, hence considerably higher exposure energies were expected based on these preliminary tests. The difference between the required and expected exposure energy from the spot test clearly shows the advantage of the step test, which is carried out based on an object from an actual printing process.

Another practical example of the step test is the investigation of a resin's absorption by using photoabsorbers. This could also be determined with the step test and would show a shift in the required exposure energy, up to the point where—due to the greatly reduced penetration depth of the UV light into the material—higher layer thicknesses such as 75–100 µm can no longer be printed in the exposure range of the DLP 3D printer. To verify this relation, 2‐nitrophenyl phenyl sulfide (NPS) was added to the home‐made resin IHB. The influence of NPS at a common photoabsorber concentration between 0.25 and 2.5 wt% was initially evaluated by spot tests, which showed an increase of *E*_c_ from 7 mJ cm^−2^ for IHB to 234 mJ cm^−2^ for IHB‐NPS2.5 and a decrease of cure depths (cf. Figure S18). In the following, the step test was performed exemplarily with 1.0 wt% NPS, which led to a considerable shift in the required exposure energy, e.g., from 75–150 mJ cm^−2^ for a 50 µm layer in case of IHB to 400–750 mJ cm^−2^ for IHB‐NPS1.0. Furthermore, the printing of 75 µm and 100 µm layers was not feasible due to photoabsorption. These tendencies could be determined just based on three 3D prints per resin using the step test (cf. Figures S19 and S20). The results of the step test can be used to identify shifts in the printable range and to reliably select suitable layer thicknesses depending on the resin. In addition to determining the printable range of a resin, the step test also provides initial information on mechanical material properties, such as flexible, stiff, or brittle, and surface properties being smooth, rough as well as (non)sticky, as well as the color of the material depending on the number of layers and their thickness. For the home‐made resins, we showed that the use of 0.2 wt% BAPO as photoinitiator resulted in a transparent and stiff material, which is advantageous for applications like microfluidics, whereas the addition of curcumin or NPS as photoabsorber led to a strong yellow coloration of the material. Apart from the color, the materials have a clear appearance which is preferred, e.g., for microscopic in situ visualization of fluid movement in microfluidic lab‐on‐a‐chip devices.

### Precise DLP 3D Printing of Polymer Micro‐ and Meso‐Structures

**Figure 5:**
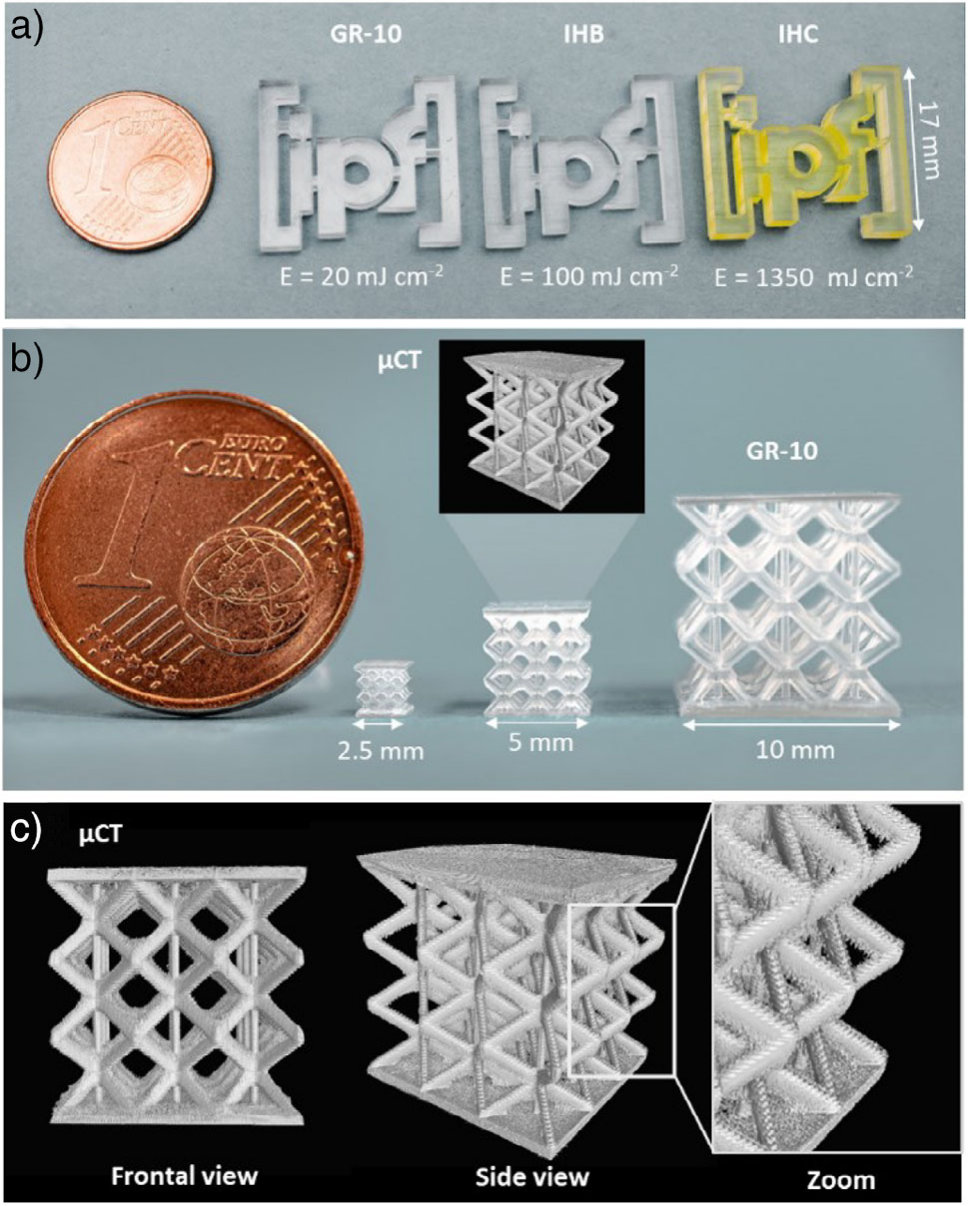
Images of a) 3D‐printed IPF logos made of the materials GR‐10 guide and home‐made resins IHB and IHC and b) precise 3D‐printed lattice structures made of GR‐10 guide in different dimension between 2.5 to 10.0 mm. c) µCT reconstruction of the 5.0 mm lattice.

Based on the determined printable range using the step test, 3D printing parameters are selected to show a precise and easy printing of objects with the commercial resin GR‐10 guide as well as the home‐made resins IHB and IHC (Figure 5). To verify the defined printing parameters, simple 3D‐printed objects such as the IPF logo were printed with 25 µm layers showing an accurate printing quality with sharp edges and smooth surface finish. In addition, the resin GR‐10 guide was used to print detailed lattice structures in three different dimensional sizes between 2.5 and 10.0 mm. The micro‐computed tomography (µCT) analysis of the 5.0 mm lattice also showed that no considerable over‐curing occurred during printing, resulting in a precisely printed object in accordance with the corresponding CAD model.

## Conclusion

We present an efficient and rapid test for determining optimum printing parameters in DLP 3D printing. It enables the screening of 24 printing parameter settings within one print while combining six exposure times for four different layer thicknesses between 25 and 100 µm at one exposure intensity. The different exposure time intervals and set intensities allow for testing resins with large chemical variety, and thus different reactivity. The method was applied to identify optimal printing conditions for three commercial resins in comparison to printing parameters provided by the manufacturers and also applied to define the printable range of two home‐made resins with rather different processing parameters. This demonstrates the universal character of our step test for a broad range of users. Although the evaluation of printing parameters in our step test is carried out via simple optical inspection by eye, it is in good agreement with reflected light microscopy analysis and SEM making the step test an easily accessible method without the need for elaborate or expensive equipment. In addition, a predominantly biobased resin was designed and investigated, with IBOA as the monomer and curcumin as the photoinitiator, both obtained from biomass of renewable raw materials. Using our novel step test, this resin was characterized in a rapid fashion, serving as an example of future resin development while enabling broader access to DLP 3D printing across disciplines. Due to its generality, the step test should be also applicable to other methods relying on liquid vat polymerization, e.g., stereolithography (SLA) and two‐photon polymerization (2PP).^[^ ^52^, ^53^, ^54^ ^]^

## Supporting Information

The authors have cited additional references within the Supporting Information. Experimental section (materials, preparation of 3D printing resins, spot tests, step tests); sample characterization (SEM measurements, reflected light microscopy measurements, µCT); CAD model of spot tests; projection, images and evaluation of spot tests and of 3D‐printed step tests of commercial and home‐made resins; reflected light microscopy images of step tests; and lateral resolution determination of 3D printed squares by SEM.

## Author Contributions

C.A. and M.V. developed the step test concept, M.V. performed 3D printing experiments, M.V. and A.K. analyzed the data. R.B. performed µCT measurements and reconstructions. M.V., A.K., and C.A. wrote the manuscript. M.V., A.K., and J.T. designed the study. J.T. was responsible for funding and supervision. All authors have read and agreed to the published version of the manuscript.

## Conflict of Interests

The authors declare no conflict of interest.

## Supporting information



Supporting Information

## Data Availability

The data that support the findings of this study are available from the corresponding author upon reasonable request.
