# Supplementary material for: Step Test for Rapid Screening of Material and Process Parameters for Resin Development in DLP 3D Printing
Source: Angew Chem Int Ed Engl. 2025 Jul 6;64(33):e202504154. doi: 10.1002/anie.202504154 (PMC12338430; doi:10.1002/anie.202504154)
Supplement: Supplementary file 1 — Supporting Information [file ANIE-64-e202504154-s001.docx]

Step Test for Rapid Screening of Material and Process Parameters for Resin Development in DLP 3D Printing

Michelle Vigogne^[a]^, Cosima Aeschbach^[a]^, Ricardo Bernhardt^[a]^, Anika Kaufmann^[a]*^, Julian Thiele^[a,b]*^

[a] M. Vigogne, C. Aeschbach, Dr. Ricardo Bernhard, Dr. Anika Kaufmann, Prof. Dr. Julian Thiele
Leibniz-Institut für Polymerforschung Dresden e.V.
Hohe Straße 6, 01069 Dresden
Email: [kaufmann@ipfdd.de](mailto:kaufmann@ipfdd.de), thiele@ipfdd.de

[b] Prof. Dr. Julian Thiele
Otto von Guericke University Magdeburg
Institute of Chemistry
Universitätsplatz 2, 39106 Magdeburg
Email: julian.thiele@ovgu.de

ORCID: Michelle Vigogne (0009-0009-8788-1026), Cosima Aeschbach (0009-0000-8246-0489), Anika Kaufmann (0009-0008-6877-4316), Ricardo Bernhardt (0000-0001-8471-9693), Julian Thiele (0000-0001-5449-3048)

**EXPERIMENTAL SECTION**

**Materials.** All chemicals were used without further purification. Phenylbis(2,4,6-trimethylbenzoyl)phosphine oxide (BAPO), Ultracur3D RG 1100 Black and Ultracur3D RG 3280 were obtained from BASF SE. Printodent GR-10 guide (385 nm) was obtained from Pro3Dure (Iserlohn, Germany). Propan-2-ol (IPA, $\geq$99.8%) and curcumin ($\geq$95 %) were received from Fisher Chemical. 1,6-bis(acryloyloxy)hexane (HDDA, >93%), isobornyl acrylate (IBOA, >99%), 2-phenoxyethyl acrylate (EGPEA), and 2-nitrophenyl phenyl sulfide (NPS) were purchased from Tokyo Chemical Industries (Zwijndrecht, Belgium).

**Resin preparation and DLP 3D printing.** Resins consisting of 15 wt% HDDA as crosslinker, 84.8 wt% IBOA as monomer, and 0.2 wt% BAPO (formulation named IHB) or curcumin (formulation named IHC) as photoinitiator (**Tab. S1**) were mixed by mechanically stirring in a VV3 Vortex mixer from VWR (Radnor, PA, USA). The curcumin-containing resin IHC was additionally sonicated for 10 min (VWR ultrasonic Cleaner USC – TH).

**Table S1.** Chemical composition of investigated resins.

|  | IBOA wt% | HDDA wt% | BAPO wt% | Curcumin wt% | NPS wt% |
| --- | --- | --- | --- | --- | --- |
| IHB | 84.8 | 15 | 0.2 | 0 | 0 |
| IHC | 84.8 | 15 | 0 | 0.2 | 0 |
| IHB-NPS0.25 | 84.675 | 14.875 | 0.2 | 0 | 0.25 |
| IHB-NPS1.0 | 84.3 | 14.5 | 0.2 | 0 | 1.0 |
| IHB-NPS2.5 | 83.55 | 13.75 | 0.2 | 0 | 2.5 |

For DLP 3D printing, two printers from ASIGA (Alexandria, Australia), namely ASIGA MAX X with an operating wavelength of 385 nm and an exposure intensity of 30 mW cm^-2^, and ASIGA MAX X with an operating wavelength of 405 nm and an exposure intensity of 66 mW cm^-2^, were used. Both printers have a theoretical lateral resolution of 27 µm with a projection field of 1,920 x 1,080 pixels. CAD models were designed with the software Autodesk Inventor 2023 and sliced using the software ASIGA Composer 1.3 (2021), which was also used to set the printing parameters.

**Spot tests.** 1.5 mL of commercial or prepared resin (**Tab. S1**) were pipetted onto a glass slide and exposed at a constant exposure intensity with different exposure times to cover an exposure energy range from 4 to 1,800 mJ cm^-2^, with the exception of IHC, where an exposure energy range from 1,200 to 7,200 mJ cm^-2^ was tested. For each exposure energy setting, three spots with a diameter of 4 mm were exposed (cf. **Fig. S1 a-b**), cleaned with IPA, and dried with compressed air. The resulting height of polymer layers was measured with a caliper gauge (TOOLCRAFT, 150 mm). This process was repeated for different exposure intensities until a stable polymer layer no longer formed due to insufficient exposure energy settings.

**Step tests.** Different layer thicknesses (z) were investigated at different exposure times (t) and exposure intensities (I) allowing for testing 24 parameter combinations (I = const., z = 25 - 100 µm, t = 2 - 12 s or t = 5 - 30 s or t = 10 - 60 s) in one print. The test object with four stairs was built up from different layer thicknesses ranging from 25 µm to 100 µm, and 3D-printed in one process. Each stair consisted of six steps, and was 3D-printed applying different exposure energies per step. For an initial estimation of the print resolution, a gap of 400 µm was integrated in-between each stair, which was used to additionally evaluate the print result. The test range for the exposure energy was studied from 4 mJ cm^-2^ (2 s at 2 mW cm^-2^) to 3,600 mJ cm^-2^ (60 s at 60 mW cm^-2^) (**Tab. S2**), which corresponds to the maximum exposure intensity of the printer ASIGA MAX X (405 nm) and provides a realistic range of exposure time per layer.

**Table S2**. Tested 3D-printing parameters for different resins.

|  | Layer thickness [µm] | Exposure intensity [mW cm^-2^] | Exposure time [s] | Exposure energy [mJ cm^-2^] |
| --- | --- | --- | --- | --- |
| Ultracur3D RG 1100 Black* | 25 - 50 - 75 - 100 | 2, 5, 10 | 2 - 12 | 4 - 120 |
| Ultracur3D RG 3280* | 25 - 50 - 75 - 100 | 1 1, 2, 5 | 1 - 6  2 - 12 | 1 - 120 |
| Printodent GR-10 guide* | 25 - 50 - 75 - 100 | 2, 5, 10, 20 | 2 - 12 | 4 - 240 |
| Formulation IHB** | 25 - 50 - 75 - 100 | 5, 10, 20 | 5 - 30 | 25 - 600 |
| Formulation IHC** | 25 - 50 | 20, 30, 45, 60 | 10 - 60 | 200 - 3,600 |
| Formulation IHB-NPS1.0** | 25 - 50 | 10, 20, 30 | 5 - 30 | 50 - 900 |

***** Baseplate printed with three 50 µm high layers at exposure energy E_6_

** Baseplate printed with three 50 µm high adhesive layers containing 83 wt% EGPEA, 15 wt% HDDA, 2 wt% BAPO at an exposure energy of 15 mJ cm^-2^.

**Sample characterization.**

**Scanning electron microscopy (SEM)**. SEM images were obtained on a NEON40EsB scanning electron microscope with a field emission cathode (Carl Zeiss Microscopy Deutschland GmbH, Oberkochen, Germany) operated at 3 kV using an Everhart-Thornley detector. For evaluating object sizes, the software Olympus Stream Desktop 2.5 (Evident Europe GmbH, Hamburg, Deutschland) was used. 3D-printed squares for lateral resolution analysis were fixed on an aluminum sample carrier with conductive silver and coated with approx. 10 nm carbon film.

**Reflected light microscopy.** Reflected light microscopy images of the step test profiles were obtained with an OLYMPUS BX51 microscope (EVIDENT Europe GmbH, Hamburg, Germany) with 5x objective. The software Olympus Stream Essentials 1.9.1 (EVIDENT Europe GmbH, Hamburg, Germany) was used to capture images and evaluate 3D-printed objects.

**Micro-computed tomography (µCT).** The μCT measurements were performed with a laboratory μCT device (CT-ALPHA, ProCon x-ray GmbH, Sarstedt, Germany) using an x-ray energy of 60 keV, and a tube target current of 110 μA. The sample was scanned along 2,500 positions in a 360-degree trajectory. Two x-ray absorption images were averaged for a single position to reduce the signal-to-noise ratio. With the CT reconstruction software X-AID (Version 2022.12.3 MITOS GmbH, Garching, Germany), a spatial image resolution of 6 μm was achieved. The interior of the 3D-printed structure was visualized using the software VG Studio MAX (Version 3.5, Volume Graphics GmbH, Heidelberg, Germany).

**Tensile test.** For mechanical measurements, tensile test dog bones (DIN 53504/S3a) were printed with RG 1100 Black using the ASIGA MAX X at 385 nm. First, a dog bone-shaped baseplate with 1 x 100 µm layers (0.1 mm) made of RG 1100 Black was exposed for 5 s with 5 mW cm^-2^ to achieve good adhesion to the print head. Afterwards, 19 x 100 µm layers (1.9 mm) were printed with the same material with 3.5 s exposure time and 5 mW exposure intensity. The resulting dog bones with a final height of 2 mm were rinsed with IPA, sonicated for 2 min in IPA, heated for 30 min at 40 °C and post-cured by UV light exposure for 2 x 45 minutes (ultraviolet lamp, type DR-301C, 36 W, 365 nm, 8 mW cm^-2^). Tensile tests in accordance with ASTM 638/S3a were carried out with a tensile test machine (ZwickRoell GmbH & Co. KG, Ulm, Germany) with a 1 kN load cell, a pneumatic sample holder (Zwick 8287, grooved metal, 5 bar), starting with 0.5 N initial load and 1 mm min^-1^ test velocity. Values for elastic modulus, elongation at break, and maximum tensile strength were determined for nine samples.

**Bending test.** For mechanical measurements, bending test samples (50x10x2 mm, in accordance to DIN EN ISO 178) were printed with RG 1100 Black using the ASIGA MAX X at 385 nm. First, a baseplate with 1 x 100 µm layers (0.1 mm) made of RG 1100 Black was exposed for 5 s with 5 mW cm^-2^ to achieve good adhesion to the print head. Afterwards, 19 x 100 µm layers (1.9 mm) were printed with the same material with 3.5 s exposure time and 5 mW exposure intensity. The resulting dog bones with a final height of 2 mm were rinsed with IPA, sonicated for 2 min in IPA, heated for 30 min at 40 °C and post-cured by UV light exposure for 2 x 45 minutes (ultraviolet lamp, type DR-301C, 36 W, 365 nm, 8 mW cm^-2^). Bending test in accordance with DIN EN ISO 178 were carried out with a universal testing maschine (ZwickRoell GmbH & Co. KG, Ulm, Germany) with a 1 kN load cell, starting with 1 N initial load and 2 mm min^-1^ test velocity. Values for flexural modulus and flexural strength were determined for eight samples.

**
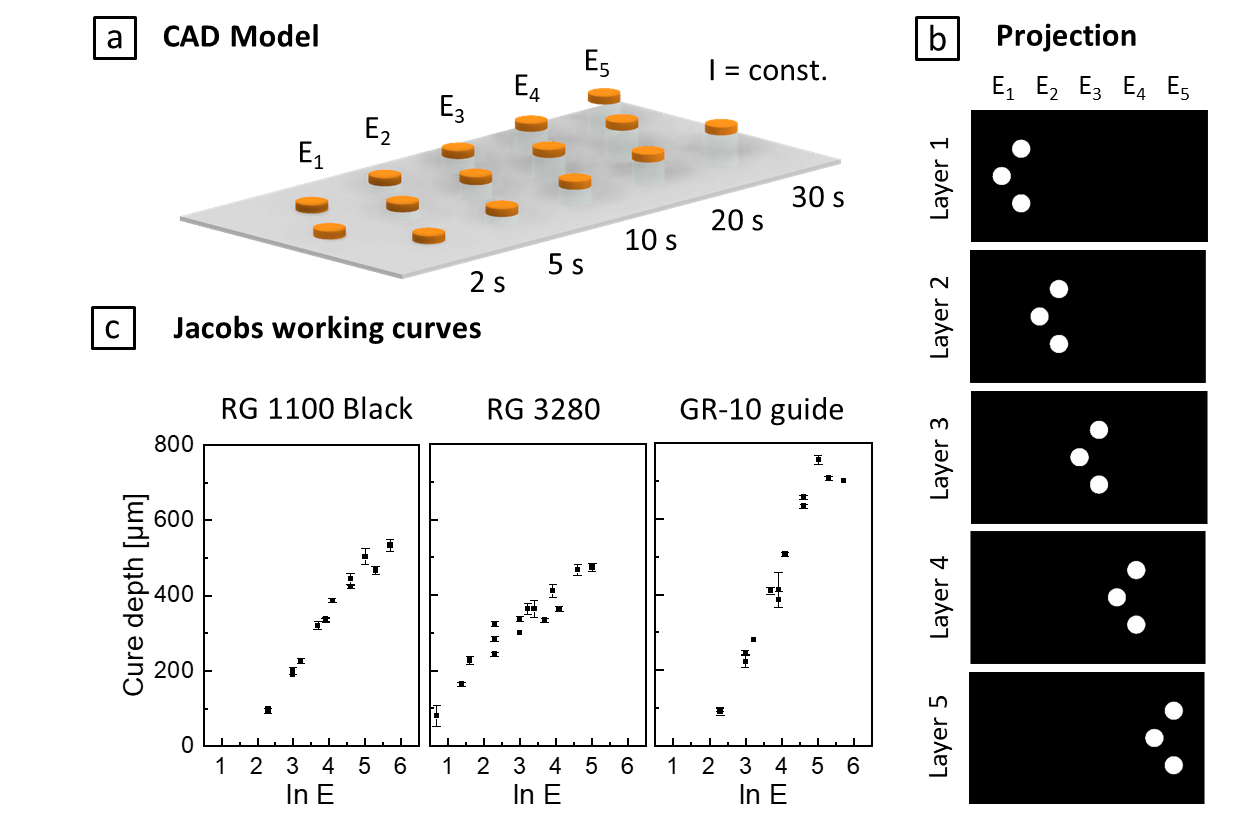
**

**Figure S1.** Spot test to determine five different exposure energies (E_1_ - E_5_) per print in triplicate, a) schematic CAD model (4-fold scaling in z-direction), b) projected layers during automated spot test, c) resulting Jacobs working curves (n = 3 ± s.d.) for three commercial resins determined from spots tests in **Fig. S2** (RG 1100 Black), **Fig. S3** (RG 3280), and **Fig. S4** (GR-10 guide).

**Table S3.** Resulting penetration depth (D_p_) and critical energy (E_c_) obtained from Jacobs working curves.

|  | RG 1100 Black | RG 3280 | GR-10 guide |
| --- | --- | --- | --- |
| D_p_ [µm] | 134 | 81 | 210 |
| E_c_ [mJ cm^-2^] | 4.4 | 0.4 | 6.2 |


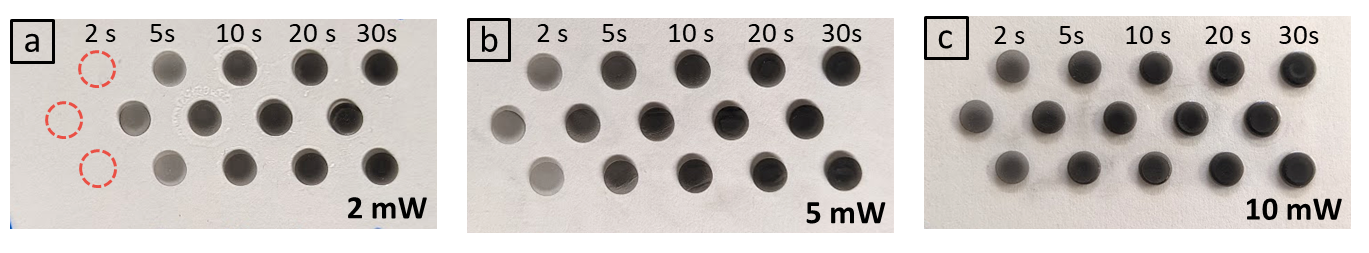


**Figure S2**. Images of spot tests of RG 1100 Black showing cured polymer layers after exposure with different exposure intensities of a) 2 mW, b) 5 mW, c) 10 mW and different exposure times at 385 nm. Dashed circles indicate the position of spots that were not printed successfully.

**
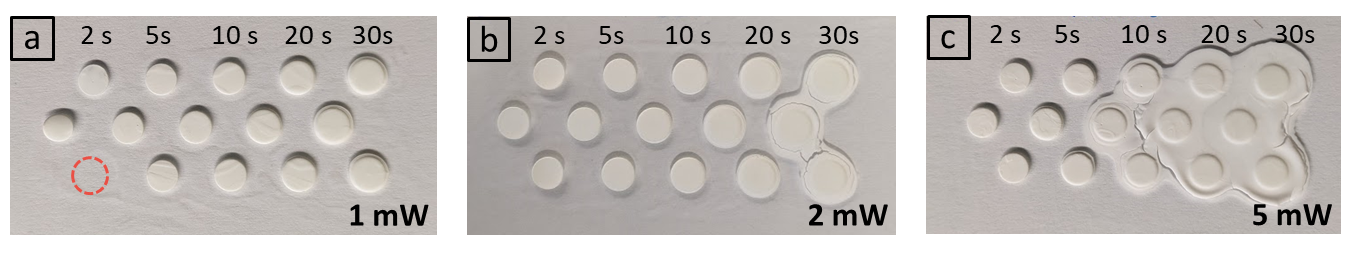
**

**Figure S3**. Images of spot tests of RG 3280 showing cured polymer layers after exposure with different exposure intensities of a) 1 mW, b) 2 mW, c) 5 mW and different exposure times at 385 nm. Dashed circle indicates the position of a spot that was not printed successfully.

**
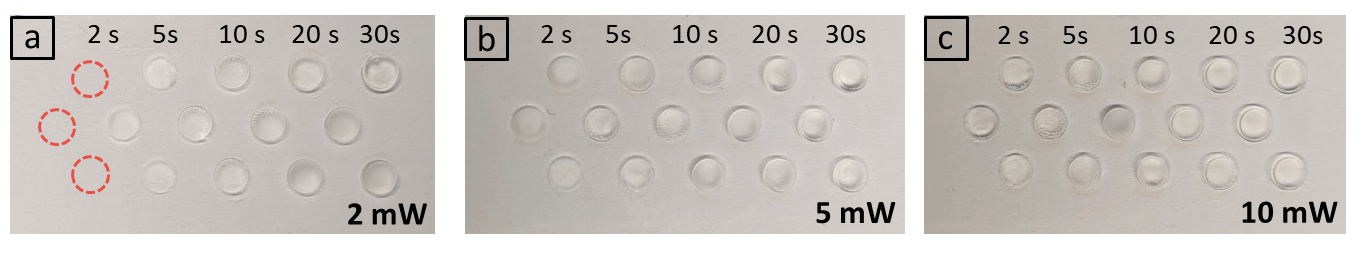
**

**Figure S4**. Images of spot tests of GR-10 guide showing cured polymer layers after exposure with different exposure intensities of a) 2 mW, b) 5 mW, c) 10 mW and different exposure times at 385 nm. Dashed circles indicate the position of spots that were not printed successfully.


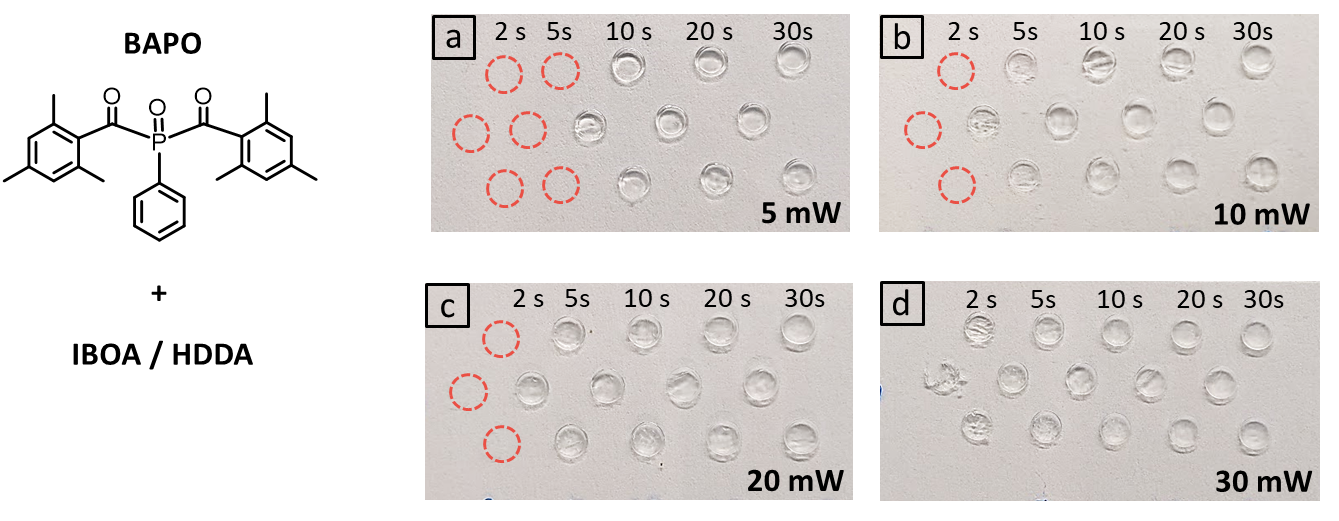


**Figure S5**. Images of spot tests of IHB (84.8 wt% **I**BOA, 15 wt% **H**DDA, 0.2 wt% **B**APO - chemical structure shown on the left side) showing cured polymer layers after exposure with different exposure intensities of a) 5 mW, b) 10 mW, c) 20 mW, d) 30 mW and different exposure times at 405 nm. Dashed circles indicate the position of spots that were not printed successfully.


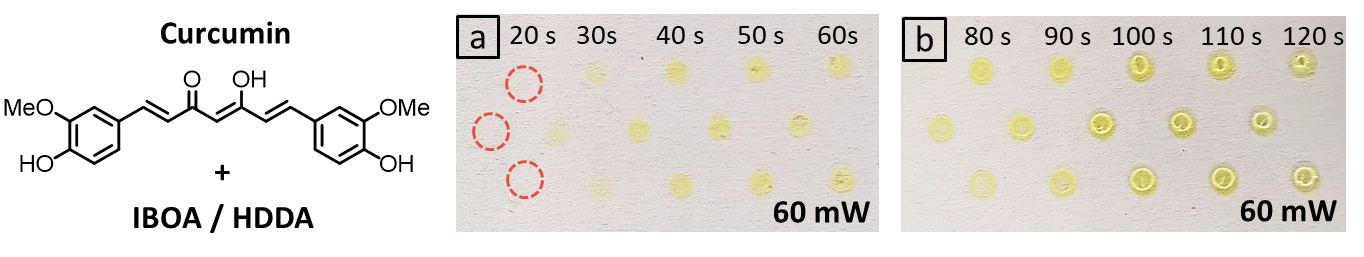


**Figure S6**. Images of spot tests of IHC (84.8 wt% **I**BOA, 15 wt% **H**DDA, 0.2 wt% **c**urcumin - chemical structure shown on the left side) showing cured polymer layers after exposure with 60 mW exposure intensity and different exposure times of a) 20 s, 30 s, 40 s, 50 s, 60 s and b) 80 s, 90 s, 100 s, 110 s, 120 s at 405 nm. Dashed circles indicate the position of spots that were not printed successfully.


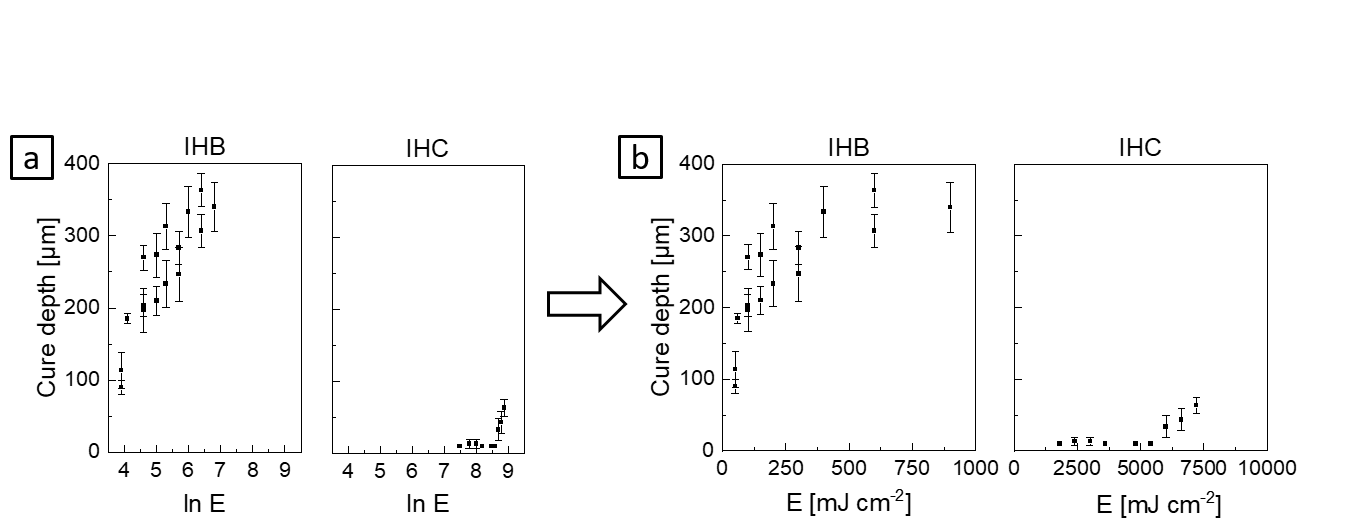
**Figure S7.** Jacobs working curves for home-made resins IHB and IHC as determined from spot tests (cf. **Fig. S5** (IHB) and **Fig. S6** (IHC)) by correlating layer thickness with a) ln E or b) exposure energy E, respectively.


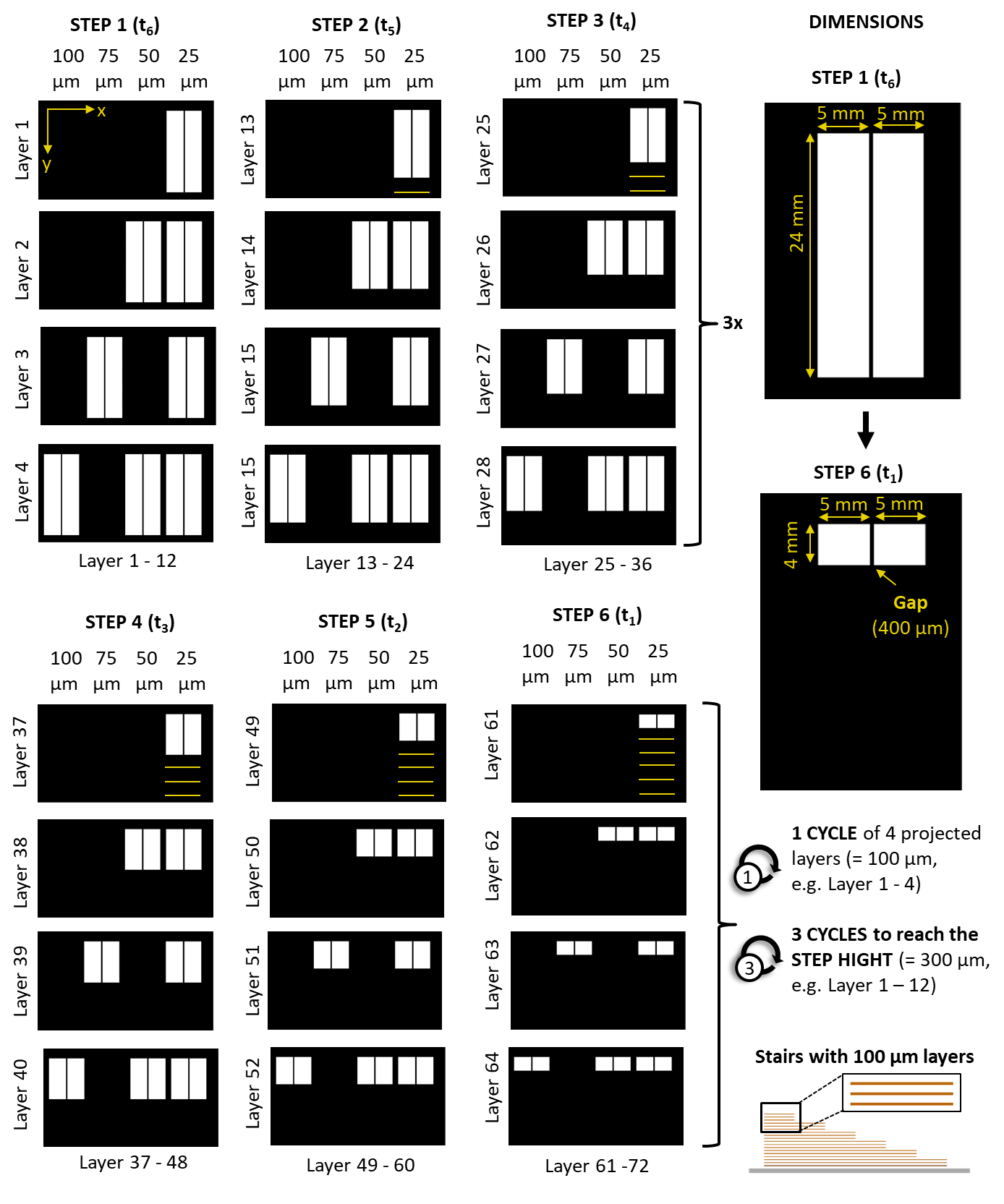


**Figure S8.** Projection for building-up the first 100 µm layers to realize six steps with each having layer thicknesses of 25, 50, 75, and 100 µm. Every step has a height of 300 µm realized by 12 x 25 µm, 6 x 50 µm, 4 x 75 µm, or 3 x 100 µm layers, respectively, resulting in 72 individual layers. Different layer heights are achieved by omitting projections. For instance, for realizing a 25 µm layer, every layer is exposed, while for a 100 µm layer only every 4th layer is exposed. Yellow lines indicate the end of the steps below. The length of the first step is 24 mm in y-direction and 10.4 mm in x-direction (divided into two 5 mm parts with an open gap of 400 µm) and decreases in size by 4 mm in y-direction with each step (step 1 to step 6: y = 24, 20, 16, 12, 8, 4 mm). The total build size of all four stairs is 24 x 46.5 x 1.8 mm (x, y, z).

**
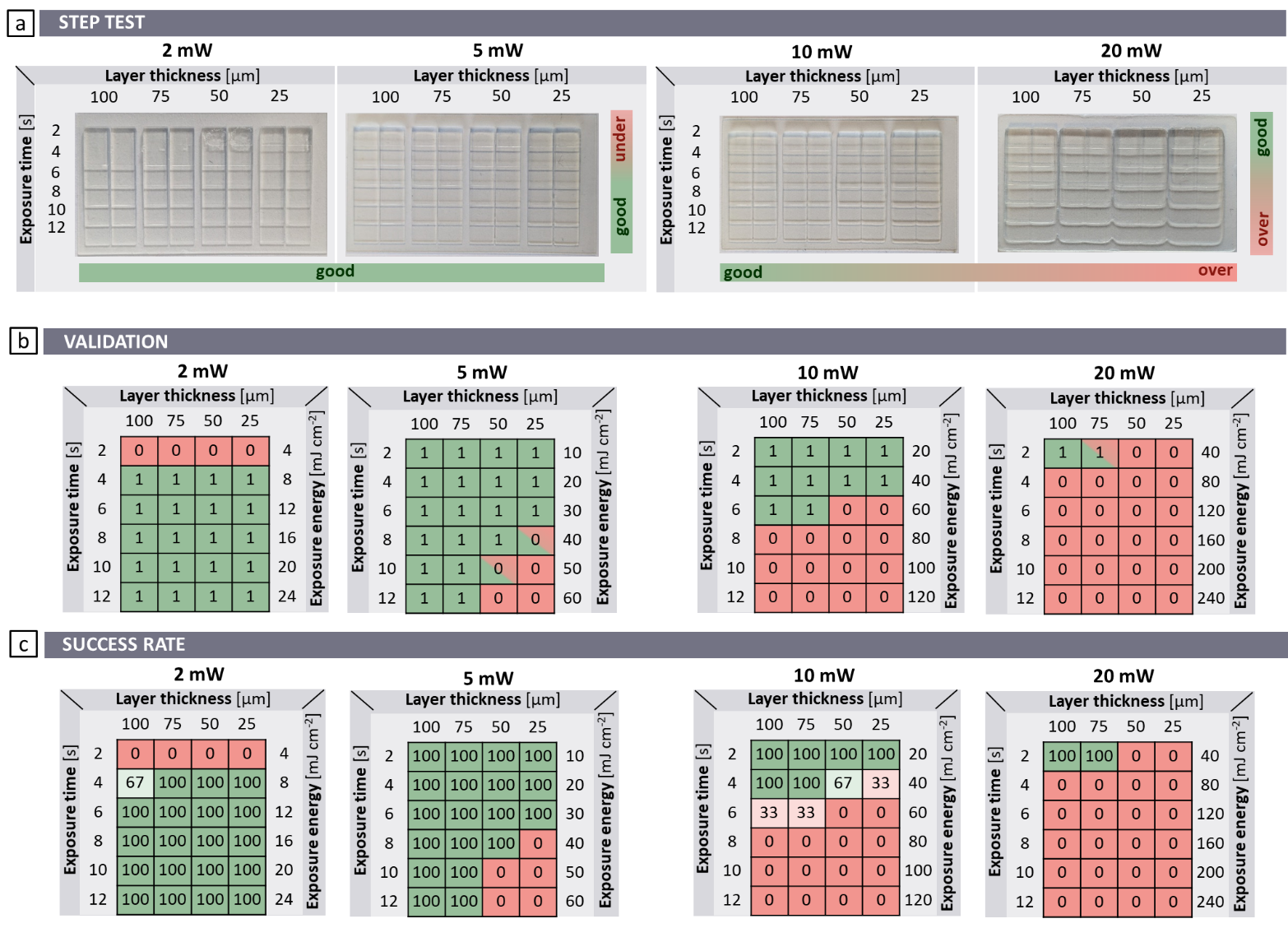
**

**Figure S9.** a) Images of step tests to determine the printable range of resin GR-10 guide at 385 nm, with step quality rated as “good”, “under” (for under-curing), or “over” (for over-curing), b) validation of individual step quality based on optical validation by eye (cf. **Fig. S9a**) and reflected-light microscopy (cf. **Fig. S11** for example images) with 0 (over-cured or under-cured) and 1 (good step quality). In case of discrepancy between both methods, the value 0 or 1 was given in favor of optical evaluation (indicated as half green - half red), c) 3-fold replication of step tests and calculation of the success rate [%] for a given printing parameter set (100% - three of three successful prints, 67% - two of three successful prints, 33% - one of three successful prints).

**Figure S10**. Alternative visualization of the step test results from **Fig. 9**: Exposure energy in correlation to exposure time and tested layer thicknesses between 25 and 100 µm for GR-10 guide at 385 nm.


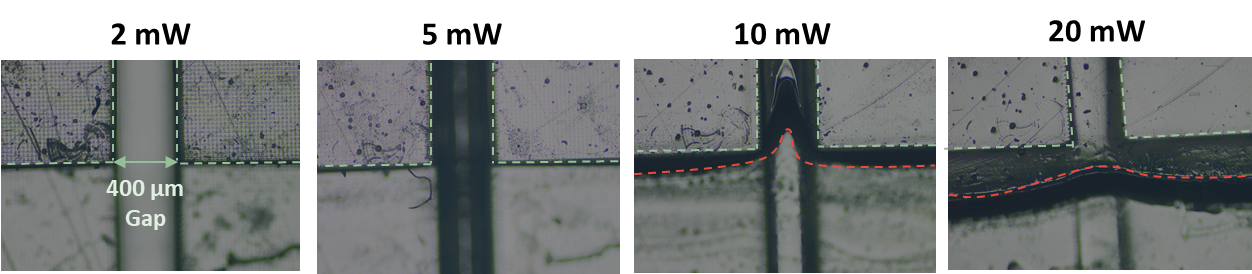


**Figure S11.** Reflected light microscopy images of steps of GR-10 guide at 2 - 20 mW exposure intensity, 6 s exposure time and 25 µm layer thickness at 385 nm (5x magnification, green line indicates step shape, red line indicates over-cured areas).


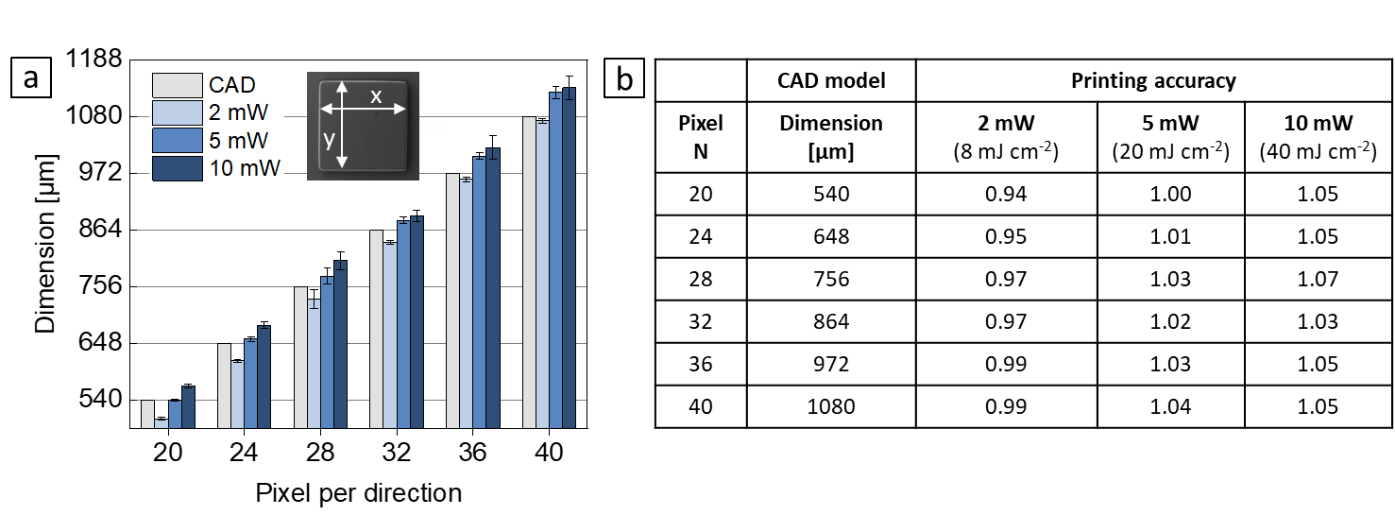


**Figure S12.** a) Dimension of squares with different pixel per direction after printing with GR-10 guide at three different exposure intensities of 2, 5, 10 mW and 4 s exposure time to build up 25 µm layers at 385 nm, and size determination with SEM (n = 6 ± s.d.) in comparison to envisioned dimensions by CAD; inset exemplary shows 1,080 x 1,080 µm (40 x 40 pixel) square printed with 10 mW exposure intensity; b) determination of printing accuracy (ratio of measured square dimensions to CAD-based square dimension).

**
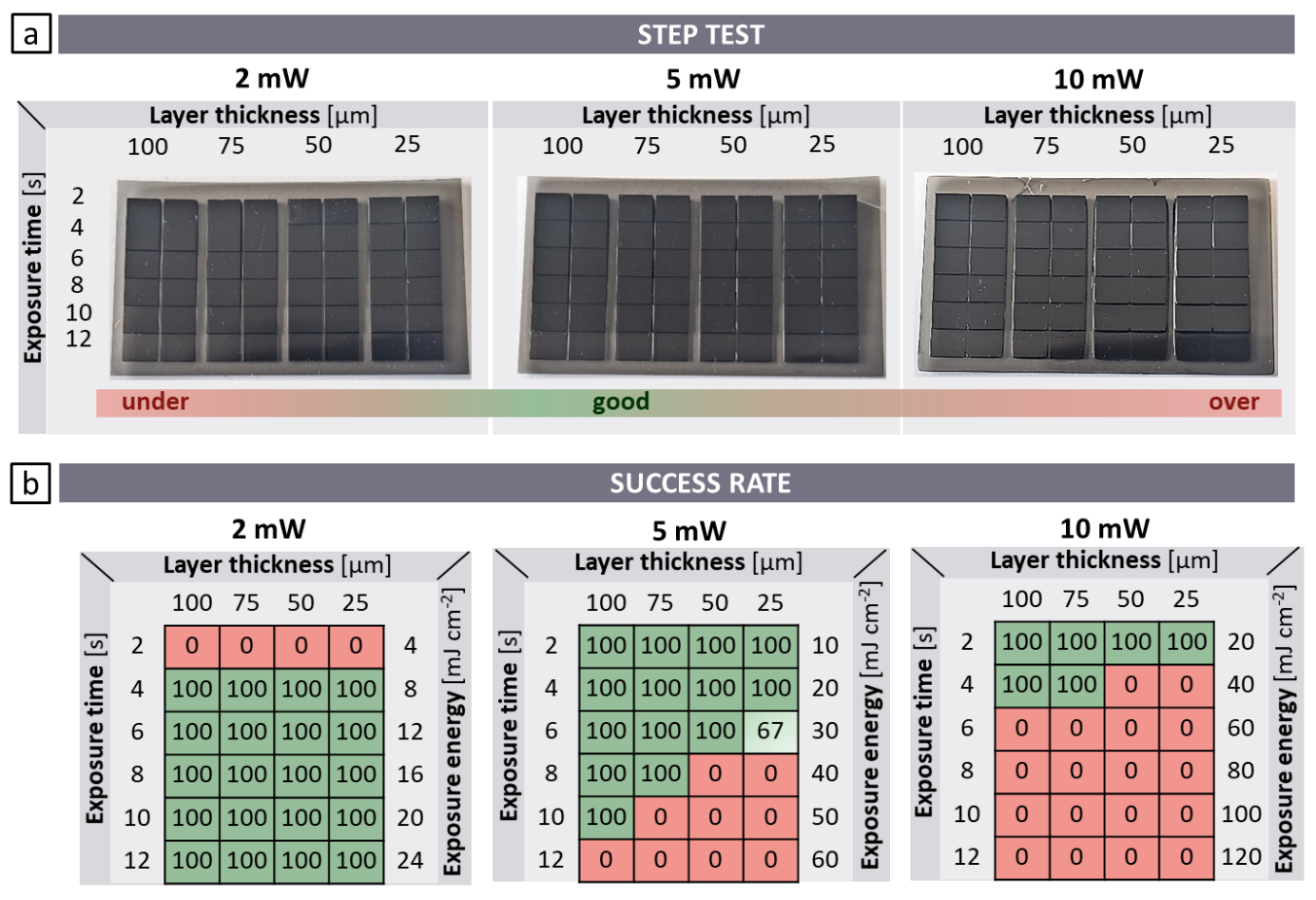
**

**Figure S13.** a) Images of step tests to determine the printable range of resin RG 1100 Black at 385 nm, with step quality rated as “good”, “under” (for under-curing), or “over” (for over-curing), b) 3-fold replication of step tests and calculation of the success rate [%] for a given printing parameter set (100% - three of three successful prints, 67% - two of three successful prints, 33% - one of three successful prints).

**Table S4.** Tensile and flexural properties of RG 1100 Black.

|  | Norm | Manufacturer’s Technical Data Sheet | Norm* | Self-printed samples** |
| --- | --- | --- | --- | --- |
| Tensile Properties |  |  |  |  |
| E Modulus | ASTM D638 | 2950 MPa | ASTMD638/S3a | 3149 ± 480 MPa |
| Tensile Strength | ASTM D638 | 70 MPa | ASTMD638/S3a | 60 ± 1 MPa |
| Elongation at Break | ASTM D638 | 5 % | ASTMD638/S3a | 6 ± 1 % |
| Flexural Properties |  |  |  |  |
| Flexural Modulus | ASTM D790 | 2790 MPa | DIN EN ISO 178 | 2707 ± 182 MPa |
| Flexural Strength | ASTM D790 | 125 MPa | DIN EN ISO 178 | 99 ± 11 MPa |

* RG 1100 Black: Printing parameters: 5 mW, 3.5 s for 100 µm, mechanical testing in accordance with the ASTM D638 and DIN EN ISO 178 test standards, but with smaller test specimens (DIN EN ISO 53505/S3a and in accordance DIN EN ISO 178/50x10x2 mm) due to the limited size of the build platform.


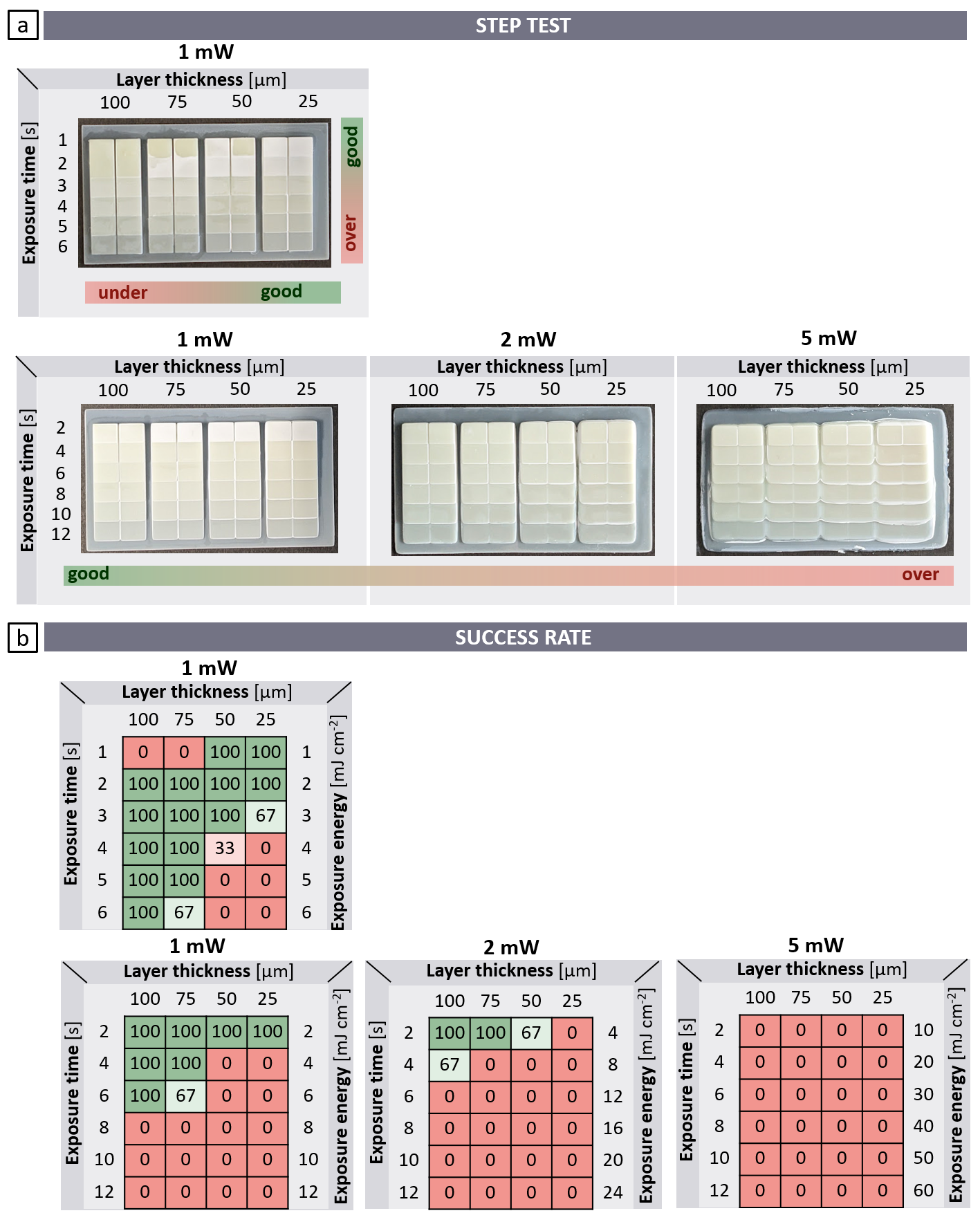


**Figure S14.** a) Images of step tests to determine the printable range of the resin RG 3280 at 385 nm, with step quality rated as “good”, “under” (for under-curing), or “over” (for over-curing), b) 3-fold replication of step tests and calculation of the success rate [%] for a given printing parameter set (100% - three of three successful prints, 67% - two of three successful prints, 33% - one of three successful prints).

**Figure S15**. Printable range and tested exposure range of two commercial resins (RG 1100 Black and RG 3280) at 385 nm, determined from images of step tests and 3-fold replication (cf. **Fig. S11** and **Fig. S12**, ideal printable range: 100% success rate, printable range: > 49% success rate of applied printing parameters).

**
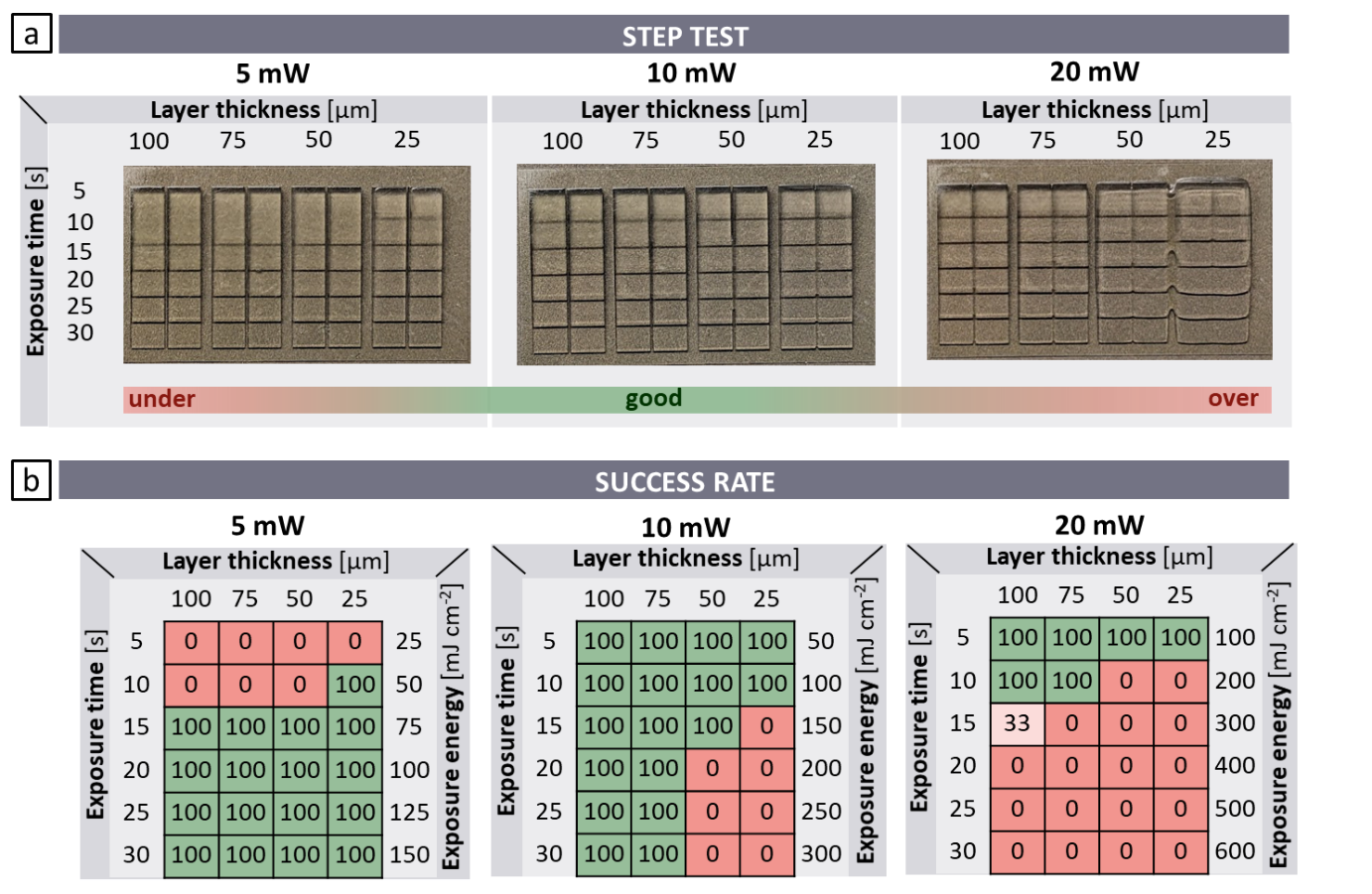
**

**Figure S16.** a) Images of step tests to determine the printable range of home-made resin IHB at 405 nm, with step quality rated as “good”, “under” (for under-curing), or “over” (for over-curing), b) 3-fold replication of step tests and calculation of the success rate [%] for a printing parameter set (100% - three of three successful prints, 67% - two of three successful prints, 33% - one of three successful prints).


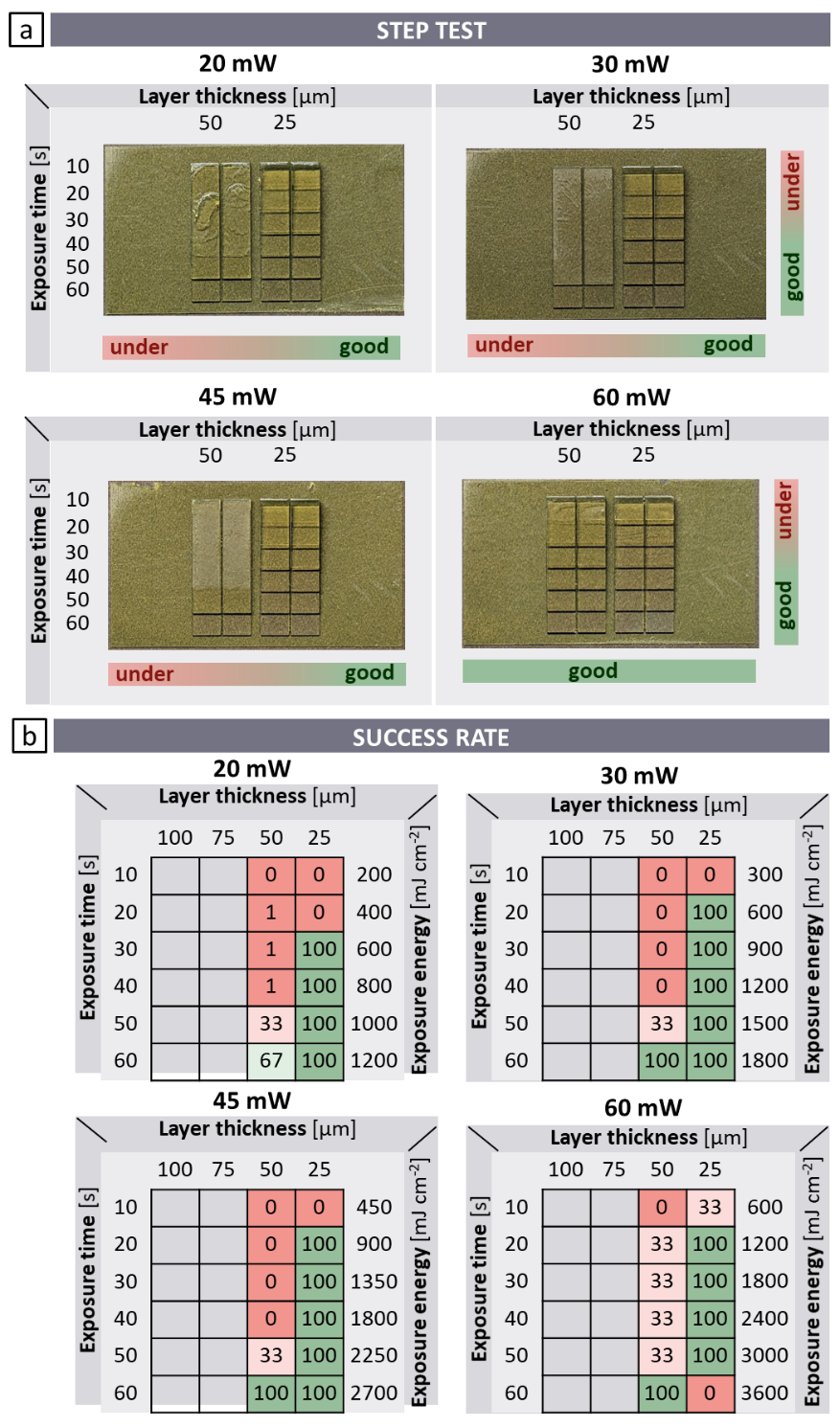


**Figure S17.** a) Images of step tests to determine the printable range of home-made resin IHC at 405 nm, with step quality rated as “good”, “under” (for under-curing), or “over” (for over-curing). Layer thicknesses of 75 µm and 100 µm were not printable, b) 3-fold replication of step tests and calculation of the success rate [%] for a given printing parameter set (100% - three of three successful prints, 67% - two of three successful prints, 33% - one of three successful prints).

**Figure S18.** Jacobs working curves of home-made resins IHB-NPS0.25, IHB-NPS1.0 and IHB-NPS2.5 (IHB resin contains 0.25, 1.0 or 2.5 wt% NPS as photoabsorber) in comparison to IHB as determined from spot tests by correlating cure depth with ln E. According to these Jacobs working curves, the following E_c_ values were determined: 7 mJ cm^-2^ (IHB), 18 mJ cm^-2^ (IHB-NPS0.25), 152 mJ cm^-2^ (IHB-NPS1.0), and 234 mJ cm^-2^ (IHB-NPS2.5).


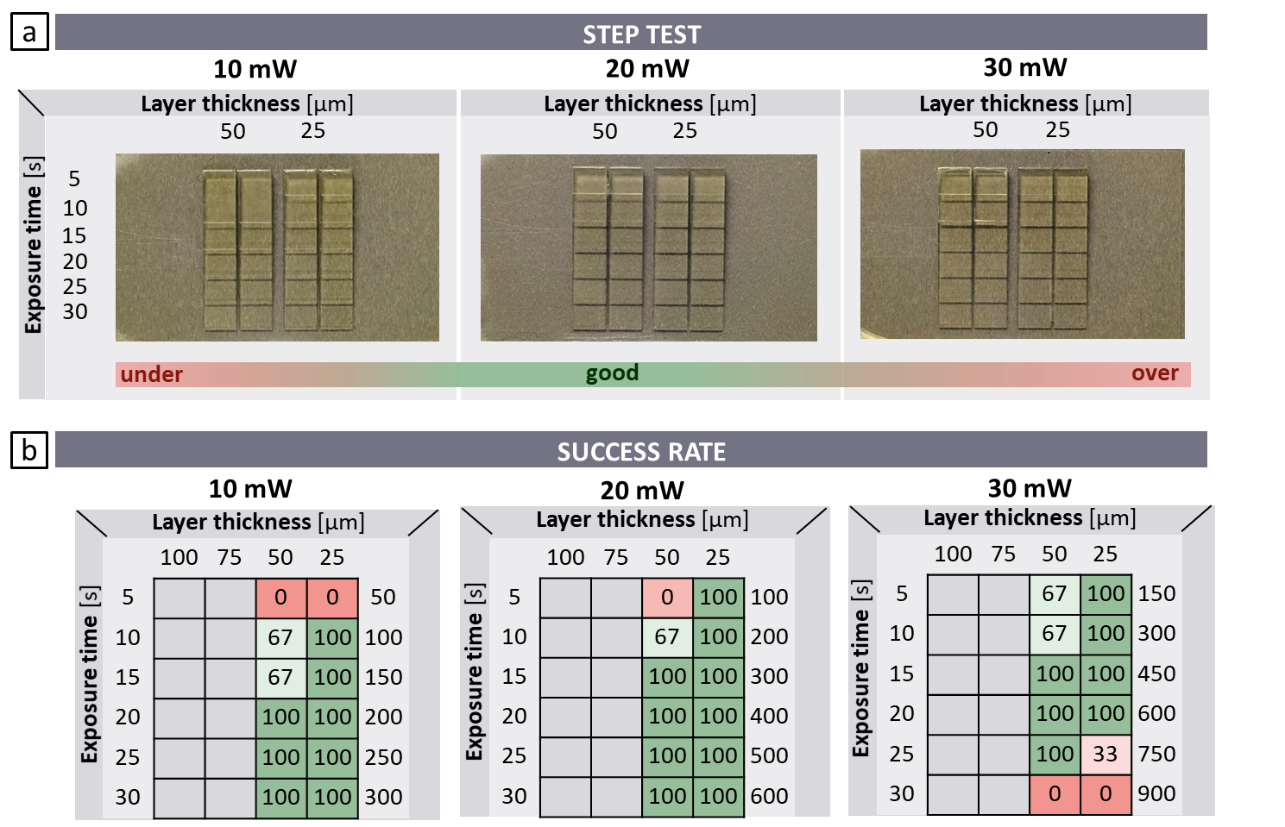


**Figure S19.** a) Images of step tests to determine the printable range of home-made resin IHB-NPS1.0 (IHB resins contains 1.0 wt% NPS) at 405 nm, with step quality rated as “good”, “under” (for under-curing), or “over” (for over-curing). Layer thicknesses of 75 µm and 100 µm were not printable, b) 3-fold replication of step tests and calculation of the success rate [%] for a given printing parameter set (100% - three of three successful prints, 67% - two of three successful prints, 33% - one of three successful prints).


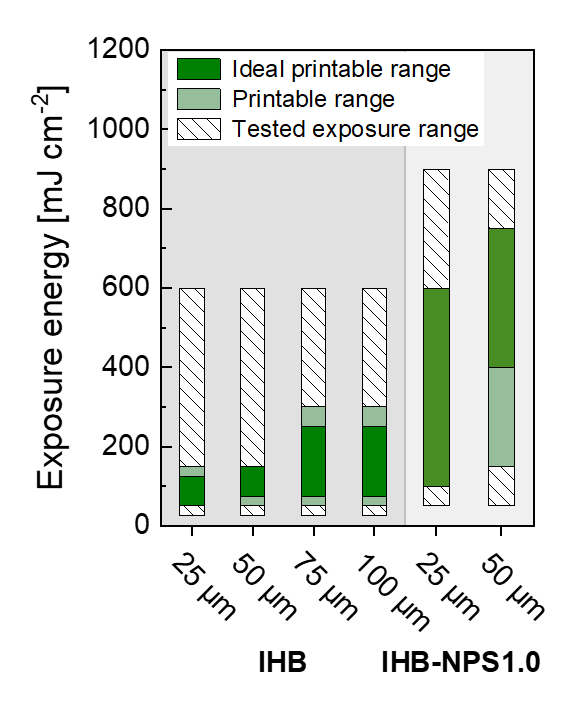


**Figure S20**. Printable range and tested exposure range of two home-made resins IHB and IHB-NPS1.0 at 405 nm, determined from images of step tests and 3-fold replication (cf. **Fig. S16** and **Fig. S19**, ideal printable range: 100% success rate, printable range: > 49% success rate of applied printing parameters).
